# Defective viral genomes as therapeutic interfering particles against flavivirus infection in mammalian and mosquito hosts

**DOI:** 10.1038/s41467-021-22341-7

**Published:** 2021-04-16

**Authors:** Veronica V. Rezelj, Lucía Carrau, Fernando Merwaiss, Laura I. Levi, Diana Erazo, Quang Dinh Tran, Annabelle Henrion-Lacritick, Valérie Gausson, Yasutsugu Suzuki, Djoshkun Shengjuler, Bjoern Meyer, Thomas Vallet, James Weger-Lucarelli, Veronika Bernhauerová, Avi Titievsky, Vadim Sharov, Stefano Pietropaoli, Marco A. Diaz-Salinas, Vincent Legros, Nathalie Pardigon, Giovanna Barba-Spaeth, Leonid Brodsky, Maria-Carla Saleh, Marco Vignuzzi

**Affiliations:** 1 Institut Pasteur, Viral Populations and Pathogenesis Unit, Centre National de la Recherche Scientifique UMR 3569, Paris, France; 2 Institut Pasteur, Viruses and RNA Interference Unit, Centre National de la Recherche Scientifique UMR 3569, Paris, France; 3 École doctorale BioSPC, Université Paris Diderot, Sorbonne Paris Cité, Paris, France; 4 Tauber Bioinformatics Research Center, University of Haifa, Haifa, Israel; 5 Institut Pasteur, Unité de Virologie Structurale, Centre National de la Recherche Scientifique UMR 3569, Paris, France; 6 Institut Pasteur, Unité de Recherche et d’Expertise Environnement et Risques Infectieux, Groupe Arbovirus, Paris, Cedex 15 France

**Keywords:** Virology, Viral infection

## Abstract

Arthropod-borne viruses pose a major threat to global public health. Thus, innovative strategies for their control and prevention are urgently needed. Here, we exploit the natural capacity of viruses to generate defective viral genomes (DVGs) to their detriment. While DVGs have been described for most viruses, identifying which, if any, can be used as therapeutic agents remains a challenge. We present a combined experimental evolution and computational approach to triage DVG sequence space and pinpoint the fittest deletions, using Zika virus as an arbovirus model. This approach identifies fit DVGs that optimally interfere with wild-type virus infection. We show that the most fit DVGs conserve the open reading frame to maintain the translation of the remaining non-structural proteins, a characteristic that is fundamental across the flavivirus genus. Finally, we demonstrate that the high fitness DVG is antiviral in vivo both in the mammalian host and the mosquito vector, reducing transmission in the latter by up to 90%. Our approach establishes the method to interrogate the DVG fitness landscape, and enables the systematic identification of DVGs that show promise as human therapeutics and vector control strategies to mitigate arbovirus transmission and disease.

## Abstract (web summary)

Defective viral genomes (DVGs) can interfere with virus replication and provide a potential approach to control infection. Here, Rezelj et al. use a combined experimental evolution and computational approach to identify DVG sequences that optimally interfere with Zika virus infection and show antiviral activity in mice and mosquitoes.

## Introduction

Viral infections give rise to degenerate forms of the viral genome, known as defective viral genomes (DVGs), as a by-product of virus replication. These DVGs manifest as genomes with complementary ends, deleterious point mutations, deletions, insertions, mosaic rearrangements, or a combination of these. As a result, DVGs cannot complete a full replication cycle. Following their discovery after serial passaging of influenza virus at high multiplicity of infection (MOI) in 1947^1^, DVGs have been described for nearly every virus family.

Although DVGs cannot self-replicate, complementation by wild-type (WT) virus enables their propagation within a virus population, by providing functions that they have lost. Certain DVGs interfere with virus replication by competing for viral and/or host resources or by enhancing immunostimulation, among other means. These are known as defective interfering particles (DIPs). While DIPs are known to be effective against some viruses in cell culture, they are largely considered a nuisance and artifact of cell culture passaging conditions. However, a rising number of recent reports describe DVGs in clinical and natural isolates: in patients infected with influenza A virus^2^, respiratory syncytial virus^3^, hepatitis C virus^4,5^, and dengue virus;^6^ in birds infected with West Nile virus;^7^ and even in plants^8,9^, suggesting that DVGs are not strictly an in vitro phenomenon. A major obstacle in exploring their potential for application as therapeutic agents for viral diseases is the difficulty in isolating DVGs with antiviral capacity from the much larger spectrum of defective genomes generated during WT virus replication.

Arthropod-borne viruses (arboviruses) pose a major global public health threat, given their continuous and rapid (re-)emergence. These viruses are maintained in nature in a cycle between invertebrate vectors and vertebrate amplification hosts. The effect of DVGs on either or both hosts has not been investigated extensively. Here, we describe a method to triage DVG sequence space to identify fit DVGs generated during virus replication in both vertebrate and invertebrate environments. Further, we demonstrate that these DVGs can exert antiviral activities in both hosts, highlighting their potential as an intervention to perturb or control arbovirus infection and transmission.

## Results

### Experimental evolution to identify Zika virus defective viral genomes (DVGs) in vertebrate and invertebrate host environments

Our primary goal was to develop a method to identify DVGs with the best potential to inhibit WT virus. While hundreds to thousands of different DVGs are generated during virus replication, we hypothesized that DVGs capable of competing and interfering with WT virus should satisfy certain criteria: occur more frequently and with greater abundance than other DVGs in conditions favoring co-infection; be observed in several replicates and in different host cell types; and be packaged into virions to infect new cells over several passages. We thus passaged Zika virus in vertebrate (Vero) and invertebrate (C6/36) cell lines at high multiplicity of infection (MOI), a condition that favors co-infection of cells by multiple genomes and thus, complementation by WT virus. We additionally performed low MOI passages to compare DVGs that arose in conditions with reduced probability of complementation with WT virus. After 12 serial passages (Supplementary Fig. 1), we sequenced encapsidated RNA genomes to identify all deletions generated in the passage series. In total, we identified 6303 and 6184 different deletions in the Vero and C6/36 high MOI passage series, respectively (Supplementary Data 1 and  2). While small deletions (1–10 nucleotides) were common in both low and high MOI conditions, larger deletions were abundant only in high MOI conditions (Supplementary Fig. 2), suggesting that larger deletions constitute competitive DVGs that rely on WT virus for propagation. It is worth noting that we did not observe a clear reduction in WT infectious virus in high MOI compared to low MOI conditions (Supplementary Fig. 1), suggesting that inhibitory DVGs did not reach high enough levels to outcompete WT virus under these growth conditions in this cell type.

### A deletion cluster with higher relative fitness is identified across multiple flaviviruses

**Fig. 1:**
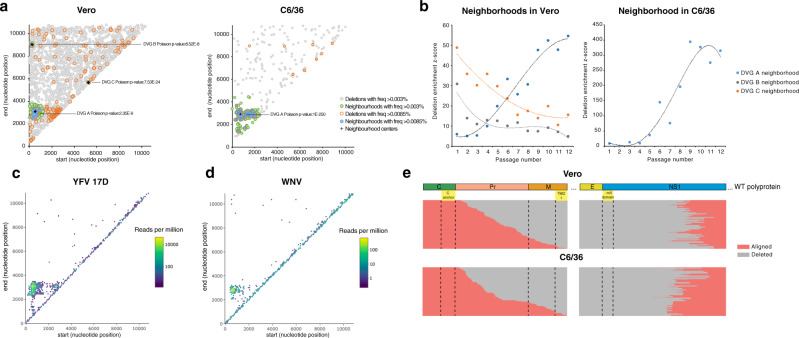
Identification and genetic characterization of Zika virus DVGs with high fitness. Virus populations enriched in DVGs, passaged in Vero or C6/36 cells, were sequenced and deletions identified. **a** Computationally-determined neighborhoods enriched with deletions in Vero or C6/36 cells are shown in a 2-dimensional plane representation of the Zika virus genome, with start and end positions of the identified deletions denoted as dots on the plane. Poisson *p* values for each neighborhood are shown. **b** Dynamics of deletion enrichment within DVG neighborhoods identified in Vero and C6/36 cells. Enrichment z-scores indicate total enrichment significance of deletions within each neighborhood from all replicates per passage. **c**, **d** Hexbin plot of deletions identified following Yellow fever virus 17D (**c**) or West Nile virus high MOI passaging in SW-13 or C6/36 cells, respectively. Identified deletions with defined start and end positions within each hexagon (>10 nt in length, bin = 100) are shown. The color of each hexagon is relative to the sum of the reads per million of deletions in all passages and replicates. For YFV, passages number 1, 8, 14, and 19 (*n* = 12) were sequenced and shown. For WNV, data from all passages and replicates (*n* = 3) are shown. **e** Putative Zika virus DVG-A genomes identified in Vero or C6/36 cells that conserve the open reading frame aligned against the Zika virus polyprotein. Aligned regions are shown in pink and deleted regions in gray. Dashed lines denote limits of proteins or relevant protein domains. Source data are provided in the Supplementary Data 1 and 2 for Vero and C6/36 data, respectively, and in Supplementary Data 3 and 4 for Yellow fever virus and West Nile virus, respectively.

To down-select DVGs with the highest potential to outcompete WT virus, we identified deletions of higher relative fitness with respect to the overall population of DVGs presenting random deletions (that is, deletions occurring with significantly increasing frequency over passages). To do this, we computationally defined regions along the Zika virus genome where deletions were most predominant using a nested neighborhood algorithm. Three neighborhoods enriched in deletions were detected in Vero cell passages (Fig. 1a, left panel) encompassing: (i) deletions from nucleotide 500 to 3500, approximately (DVG-A) (ii) large deletions stretching from the beginning of the genome to nearly the end (DVG-B); and (iii) a small deletion around nucleotide position 5500 (DVG-C). In mosquito cells, only the DVG-A neighborhood was identified as significantly enriched with deletions (Fig. 1a, right panel). Deletions were uniformly distributed within each neighborhood (Supplementary Fig. 3). Enrichment z-scores then allowed us to estimate the significance of deletions within the neighborhood, across passages. The DVG-A neighborhood exhibited increasing enrichment z-scores over the passage series in Vero and C6/36 cells, whereas the other two showed the opposite effect (Fig. 1b). Thus, deletions in the DVG-A neighborhood were predicted as fit, whereas those in DVG-B and DVG-C neighborhoods were less fit. Importantly, the deletion hotspot corresponding to the DVG-A neighborhood was exclusively found in replicates performed at high, but not low, MOI conditions, with increased frequency compared to other deletions (Supplementary Figs. 4 and 5). Once this deletion hotspot appeared, it was maintained in subsequent passages, confirming propagation from one passage to another. By contrast, deletions corresponding to neighborhoods B and C were transient, found to appear in some passages and disappear in later ones. We next asked whether this type of DVG was unique to Zika virus, or was shared among the flaviviruses. We thus performed similar passaging experiments with two other flaviviruses, yellow fever virus (Fig. 1c) and West Nile virus (Fig. 1d) and found this same neighborhood of deletions in both cases.

### High fitness DVG-A conserves the open reading frame following the deletion site

We evaluated the effect that DVG-A-type deletions inflicted on the remainder of the polyprotein still encoded in its sequences. We further delineated the deletion hotspot for both Vero and C6/36 cell lines with boundaries encompassing deletions with the highest frequencies (suggesting higher biological relevance): where the breakpoint was between nucleotide positions 500 and 900, and the rejoin was between nucleotide positions 2800 and 3400 (Supplementary Fig. 6a, d). Deletions outside the delineated region were randomly generated with respect to maintaining the open reading frame (ORF) following the deletion or frame-shifting. Strikingly, nearly 100% of deletions within the high-fitness hotspot conserved the ORF (Supplementary Fig. 6g). This observation was further supported by the yellow fever and West Nile virus data, for which 99.5% and 98.2% of deletions also conserved the ORF downstream of the deletion (Supplementary Fig. 6b, c, e, f, g).

To understand if the resulting DVG-A polyprotein retained relevant signal peptides or domains required for correct processing, translocation, and topology in the ER membrane, we aligned each putative DVG within this hotspot with the viral polyprotein (Fig. 1e). For the 244 or 418 deletions identified in Vero or C6/36 cells, respectively, every deletion followed the capsid anchor that directs the translocation of Pr into the ER lumen. Concomitantly, these deletions ended at the N-terminus of NS1, which is also found in the lumenal side of the ER. Thus, although the complete E protein, part of PrM, and NS1 are missing, the topology of the rest of the polyprotein is presumably maintained in these DVGs. Moreover, all deletions lack the β-roll domain of NS1, essential for its dimerization and function^10,11^. Taken together, our analysis suggests that while other non-structural proteins would be properly translated and processed, the truncated NS1 protein of these DVGs would be non-functional.

### DVG-A is non-replicative per se and relies on WT virus NS1 protein for genome replication

**Fig. 2:**
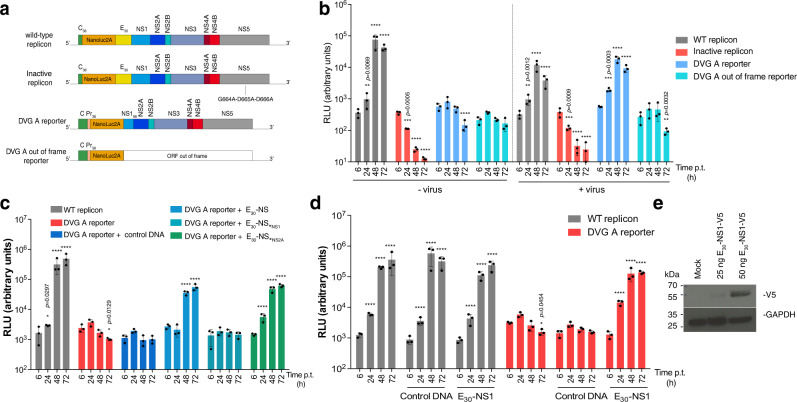
Zika virus DVG-A relies on WT-encoded NS1 for its genome replication. **a** Schematic diagram of reporter constructs. For the wild-type replicon, C_38_ and E_30_ represent the N-terminal 38 aminoacids of C and the C-terminal 30 aminoacids of E proteins, respectively. For the DVG-A replicon, Pr_38_ and NS1_98_ represent the N-terminal 38 aminoacids and the C-terminal 98 aminoacids of Pr and NS1 proteins, respectively. NanoLuc2A represents the NanoLuc reporter sequence followed by the foot-and-mouse disease virus 2A protease. An inactive replicon (with a mutated polymerase active site) was also constructed. **b** Replicon assays in infected or uninfected cells. Equal amounts of replicon or reporter RNA was transfected in naïve or infected Vero cells (MOI 1 PFU/cell), and relative light units (RLU) measured at the indicated times post-transfection. WT and inactive replicons were used as controls. ****p ≤ 0.0001. **c** DVG-A RNA reporter activity in HEK-293T cells pre-transfected with control DNA, E_30_-NS, E_30_-NS_StopNS1_ or E_30_-NS_StopNS2A_. WT replicon was used as a control. RLU were measured at the indicated times p.t. ****p ≤ 0.0001. **d** WT and DVG-A reporter assays in HEK-293T cells pre-transfected with control DNA or E_30_-NS1. RLU were measured at the indicated times p.t. ****p ≤ 0.0001. In **b**, **c**, and **d**, reporter activity was compared to that at 4 h by two-way ANOVA with Dunnet’s multiple comparison. **e** Cells transfected with a V5-tagged version of the E_30_-NS1 were harvested 24 h p.t. for western blotting, a representative blot of two is shown. Corresponding uncropped images of the western blot are shown in Supplementary Fig. 12. All graphs show the mean and SD; *n* = 3 per group for a representative experiment of three. Control DNA: GFP-expressing plasmid. Source data are provided as a Source Data file.

The ubiquity and high fitness of this DVG neighborhood led us to select DVG-A as the optimal candidate to be used as a viral competitor in downstream experiments. While some genomes containing deletions can be defective in producing their own structural proteins, they may retain their ability to self-replicate, and would thus be classified as replicons. Replicons are often used as surrogates to study replication of positive-sense RNA viruses, including Zika virus, because they retain all non-structural functions required to copy themselves and need WT virus only to encapsidate their progeny genomes^12,13^. We thus evaluated if DVG-A was either a true replicon or a truly defective genome. We genetically engineered DVG-A to represent one of the most abundant deletions identified in the DVG-A high fitness neighborhood. We also engineered a cognate DVG in which the ORF following the deletion was disrupted by removing a nucleotide at the deletion junction (hereafter referred to as DVG-A_out-of-frame). A NanoLuc reporter gene was inserted into each construct to measure genome replication by luminescence. As a positive control, we employed a WT replicon that can self-replicate and as a negative control, an inactive version of this replicon in which the catalytic motif at the polymerase active site was mutated (Fig. 2a). While the WT replicon was replication-competent on its own, the inactive replicon, DVG-A, and DVG-A_out-of-frame did not replicate (Fig. 2b, left side of panel). Importantly, DVG-A replication could be rescued in infected cells (Fig. 2b, right side of panel). Thus, DVG-A is not a true replicon, as it requires the presence of WT virus to replicate. DVG-A_out-of-frame did not replicate even in infected cells, indicating that preserving the ORF after the deletion is an absolute requirement for DVG-A genome replication.

Since DVG-A carries intact non-structural sequences except for NS1, we investigated whether this protein rescues DVG-A replication through trans-complementation. We assessed DVG-A genome replication in cells pre-transfected with plasmids encoding the non-structural proteins: namely a plasmid encoding all of the non-structural proteins, from NS1 through NS5 (E_30_-NS); a control plasmid in which a stop codon was placed in lieu of the first amino acid of NS1, such that no non-structural proteins are expressed (E_30_-NS_*NS1_); and a third plasmid in which a stop codon replaced the first amino acid of NS2A, so that only NS1 protein is expressed (E_30_-NS_*NS2A_). Only cells expressing the entire non-structural proteins or the single NS1 protein could support DVG-A replication (Fig. 2c). Thus, DVG-A relies solely on WT virus-encoded NS1 function to facilitate its genome replication. Indeed, DVG-A replication was rescued in cells transfected with a plasmid encoding only the NS1 sequence (E_30_-NS1), devoid of any downstream NS sequences that were present in the previous experiment (Fig. 2d). The expression of NS1 protein was confirmed by western blot with an antibody against a V5 epitope used to tag NS1 in the plasmid construct (Fig. 2e).

### DVG-A inhibits WT Zika virus replication in vertebrate cells through competition for cellular resources

**Fig. 3:**
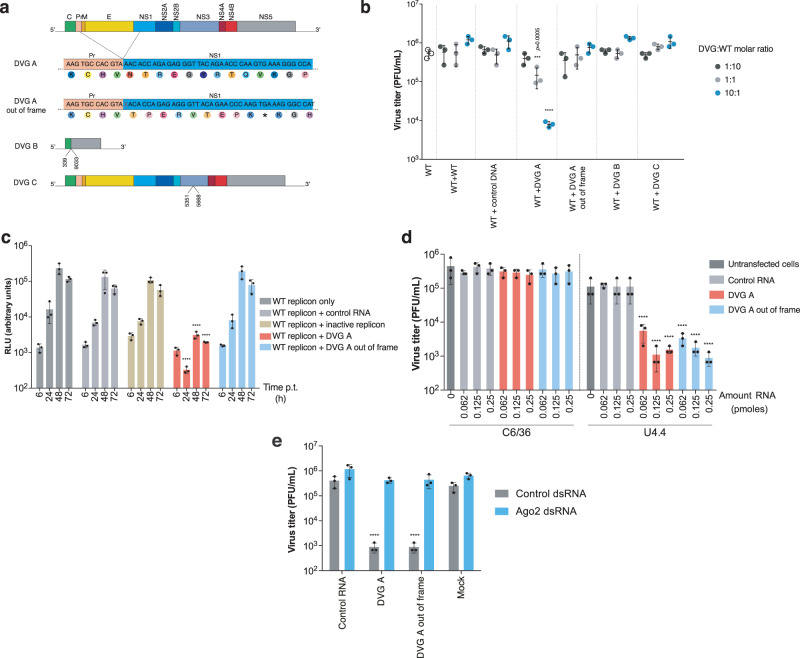
DVG-A inhibits WT virus production in vertebrate and invertebrate cells. **a** Schematic diagram of the engineered DVGs. For DVG-A and DVG-A out-of-frame, the respective nucleotide and amino acid sequences are shown. Symbol (*) refers to a stop codon. The deleted nucleotide in DVG-A out-of-frame is crossed in red. For DVG-B and DVG-C, the nucleotides at the deletion junction are indicated. **b** Virus titers 72 h following co-transfection of DVG and WT-encoding plasmids in HEK-293T cells at different DVG:WT molar ratios (1:10, 1:1, and 10:1) are shown. More WT-encoding plasmid or control DNA were used as controls. ****p ≤ 0.0001 (compared to WT + WT conditions by two-way ANOVA with Dunnet’s multiple comparison). **c** Vero cells (commonly used for replicon assays) were transfected with WT replicon RNA complemented with control RNA, inactive replicon, DVG-A or DVG-A out-of-frame. Replicon activity was measured at the indicated time points p.t. ****p ≤ 0.0001 (compared to RLU values in WT replicon only-transfected cells, by two-way ANOVA with Dunnet’s multiple comparison). **d** Inhibition assays in mosquito cells. C6/36 (left) or U4.4 (right) cells were transfected with the indicated amount of control RNA, DVG-A or DVG-A out-of-frame. 18 h later, cells were infected with a MOI of 0.1 PFU/cell and virus titers measured 5 days p.i. ****p ≤ 0.0001 (compared to untransfected cells by two-way ANOVA with Dunnet’s multiple comparison). **e** Inhibition assays in cells knocked down for Ago-2 expression. U4.4 cells were transfected with Ago-2 or control dsRNA and the indicated RNA. 18 h later, cells were infected at an MOI of 0.1 PFU/cell and virus titers measured at 5 days p.i. ****p ≤ 0.0001 (compared to control RNA-transfected cells by two-way ANOVA with Dunnet’s multiple comparison). All graphs show the mean and SD; *n* = 3 per group for a representative experiment of three. Control RNA: pTRI-Xef RNA; control DNA: GFP-expressing plasmid. Source data are provided as a Source Data file.

Our primary goal was to identify DVGs of high fitness that could best compete with WT virus. To evaluate this, we measured WT virus production in the presence of DVG. In addition to DVG-A and DVG-A_out-of-frame, we included two other DVGs representing deletion neighborhoods with lower fitness: DVG-B and DVG-C (Fig. 3a). DVG-A significantly decreased WT virus titers in a dose-dependent manner by up to 2.2-log (Fig. 3b). Interestingly, both DVG-B and DVG-C failed to inhibit WT virus production even at the highest DVG:WT molar ratio and no inhibition was observed when DVG-A_out-of-frame was used. Since DVG-A is missing the structural proteins, we further examined whether inhibition was related to competition for structural proteins provided by WT virus. To address this, we evaluated whether DVG-A could also inhibit a WT replicon lacking structural proteins. Indeed, DVG-A RNA significantly decreased WT replicon activity (Fig. 3c) and this effect was dose-dependent, with pronounced inhibition at the highest DVG:WT replicon RNA molar ratios (Supplementary Fig. 7a). Finally, since DVGs of other viruses, such as the copyback DVGs of negative-sense RNA were shown to inhibit WT virus replication by stimulating innate immune responses^3,14^, we measured the activation of three antiviral genes in cells harboring DVG-A and WT virus at a 10:1 molar ratio. No significant differences in the expression of interferon-alpha, interferon-lambda, or RIG-I were found (Supplementary Fig. 7a–c), indicating that DVG-A is no more immunostimulatory than WT Zika virus.

Thus, in mammalian cells, DVG-A seemingly inhibits WT virus production by entering in competition with WT virus for cellular machinery and resources required for genome replication, rather than by activation of innate immunity. These results also suggest that the observed inhibition requires the expression of the replicase and non-structural proteins directly by the DVG itself, once NS1 has been provided *in trans* by WT virus.

### DVG-A inhibits WT Zika virus in invertebrate cells in an RNAi-dependent manner, regardless of whether the remaining ORF is in frame

To determine if DVG-A could also inhibit Zika virus in the invertebrate host environment, we infected C6/36 mosquito cells harboring either DVG-A, DVG-A out-of-frame, or control RNA. We observed no reduction of virus titer for any treatment condition (Fig. 3d, left side of panel). Since C6/36 cells are known to have a defective Dicer-2 activity and they cannot initiate the RNA interference (RNAi) antiviral response^15,16^, we repeated the experiment in Dicer-2 competent U4.4 mosquito cells^17,18^. In this case, both DVG-A and DVG-A_out-of-frame elicited a significant decrease in virus titers (up to 2-log) compared to control conditions (Fig. 3d, right side of panel). Since RNAi is a sequence-dependent silencing mechanism, both DVG-A and DVG-A_out-of-frame inhibited virus replication, as a single nucleotide change in the DVG sequence should not prevent the mounting of an efficient RNAi response. Knockdown of Argonaute-2 (Ago-2, an essential component of the RNAi response) in U4.4 cells (up to 90% knock-down efficiency, Supplementary Fig. 7e) abrogated the inhibition of Zika virus infection (Fig. 3e). Therefore, DVG-A-induced inhibition of virus replication in the invertebrate host environment is governed by the RNAi response.

### DVG-A can be packaged into virus-like particles (VLPs) for in vivo delivery

**Fig. 4:**
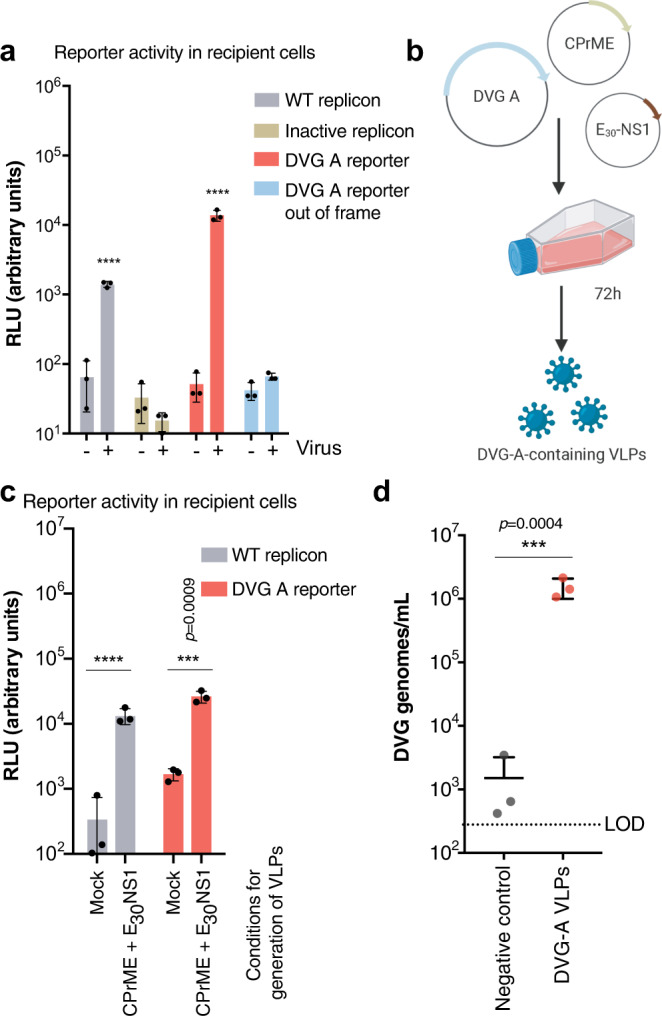
Generation of DVG-A-carrying virus-like particles. **a** Assessment of DVG-A packaging ability by WT virus. Infected or uninfected Vero cells were transfected with WT replicon, inactive replicon, DVG-A reporter, or DVG-A out-of-frame reporter RNA. 48 h post-transfection, the cell supernatant was collected from donor cells, depleted of naked RNA, and used to infect naïve recipient cells, which were harvested 24 h later for measuring reporter activity. Luciferase activity in recipient cells is shown. ****p ≤ 0.0001 (by two-way ANOVA with Dunnet’s multiple comparison, as compared to uninfected cells). **b** Schematic illustration of VLP assays: HEK-293T cells were transfected with a CPrME, E_30_-NS1 and DVG -encoding plasmids. 72 h post-transfection the cell culture supernatant with DVG-containing VLPs was harvested. **c** VLP assays using WT replicon and DVG-A reporter. Donor HEK-293T cells were transfected with WT replicon or DVG-A reporter plasmid, with or without (mock) CPrME and E_30_-NS1. The cell culture supernatant was treated with nuclease and used to infect naïve recipient Vero cells. Reporter activity in recipient cells is shown. ****p ≤ 0.0001 (by two-way ANOVA with Dunnet’s multiple comparison, as compared to mock conditions). **d** Quantification of DVG-A genomes in VLPs generated using native DVG-A. Clarified and nuclease-treated supernatant from donor cells were subjected to DVG-A quantification using RT-qPCR. Negative control refers to supernatant derived from cells transfected with the same amount of DVG-A-containing plasmid in the absence of CPrME or E_30_-NS1. ***p = 0.0004 (by two-tailed t-test). All graphs show the mean and SD; *n* = 3 per group of a representative experiment out of three. Source data are provided as a Source Data file.

To continue exploring DVG-A as a potential therapeutic agent, we required an appropriate delivery method. Thus, we established a packaging system to encapsidate DVG-A into virus-like particles (VLPs). We first needed to confirm that DVG-A could be encapsidated at all. To this end, we transfected uninfected or infected Vero cells with DVG-A reporter or control WT replicon RNA. These cells, named ‘producer’ cells, should (if infected) produce WT virus progeny, as well as virions containing genomes that can be packaged. Transfer of the supernatant derived from infected producer cells to naïve (recipient) cells enabled us to assess packaging of reporter-expressing genomes. Significantly higher WT replicon and DVG-A reporter activities were observed in recipient cells in the presence of WT virus, compared to background activity in the absence of virus, confirming that DVG-A was packaged by WT virus (Fig. 4a, producer cell reporter activity is shown in Supplementary Fig. 8a). Comparatively, no increase in reporter activity was detected for the inactive replicon or DVG-A_out-of-frame, suggesting that genome replication is a requirement for genome packaging.

Next, we developed a VLP-producing system to encapsidate DVG-A in the absence of WT virus. Briefly, we transfected HEK293T cells (chosen due to their high transfection efficiency) with plasmids encoding DVG-A, the structural proteins (CPrME), and the non-structural protein NS1, to enable packaging and replication of DVG-A, respectively (Fig. 4b). Packaging of DVG-A into VLPs was then assessed following transfer of supernatants onto naïve recipient Vero cells. Using reporter DVG-A, we confirmed that the reporter activity in recipient cells for packaged DVG-A was comparable to that of packaged WT replicon (Fig. 4c, producer cell reporter activity shown in Supplementary Fig. 8b). Finally, we confirmed that native DVG-A was packaged into VLPs, as shown by RT-qPCR of transfected cell supernatants (Fig. 4d). Importantly, we confirmed by plaque assay that no infectious virus was produced with the VLPs (Supplementary Fig. 9).

Thus, DVG-A can be actively packaged into VLPs, which could serve as therapeutic interfering particles (TIPs) for in vivo evaluation.

### Zika virus TIPs reduce infection and virulence in mice

**Fig. 5:**
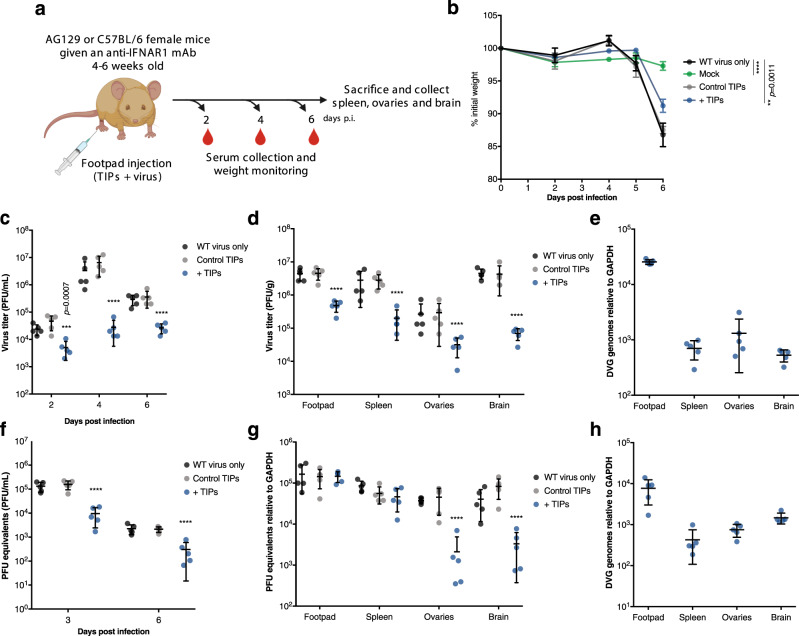
Zika virus DVG-A-carrying TIPs inhibit virus replication in mice. **a** Experimental design. 4–6-week-old AG129 or C57BL/6 female mice were inoculated with WT virus diluted in DMEM, mock TIP supernatant (from transfections performed with no structural proteins), or with supernatant confirmed to contain DVG-packaging VLPs (TIPs). C57BL/6 mice were treated with 2 mg of IFNAR1-blocking monoclonal antibody 24 h prior to infection. At different times post-infection, sera were collected. 6 days p.i. mice were euthanized and organs harvested. **b**–**e** AG129 mice: (**b**) Weight loss of infected mice is shown as a % of weight at day 0 (*n* = 5). ****p ≤ 0.0001. **c** Viremia on days 2, 4, and 6 post infection (*n* = 5). ****p ≤ 0.0001. **d** Virus load in the injected footpad, spleen, ovaries and brain, 6 days p.i. (*n* = 5) ****p ≤ 0.0001. **e** DVG amounts in all organs collected measured by RT-qPCR (*n* = 5). **f**–**h** C57BL/6 female mice that were given an IFNAR1-blocking monoclonal antibody 24 h prior to infection. **f** 3 and 6 days p.i. viremia was measured by RT-qPCR and is depicted as PFU equivalents/mL (*n* = 5). ****p ≤ 0.0001. **g** 6 days p.i. mice were euthanized and virus load in organs measured by RT-qPCR (*n* = 5). ****p ≤ 0.0001. **h** DVG-A amounts in all organs measured by RT-qPCR (*n* = 5). All graphs show the mean and SD; *n* = 5. In all cases, two-way ANOVA with Dunnet’s multiple comparison was carried out comparing each condition to mice infected with WT virus only. Source data are provided as a Source Data file.

We next assessed the efficacy of these TIPs as an antiviral measure in mice. We used mice deficient in α/β and γ receptors (AG129 mice), as these are highly susceptible to Zika virus infection and disease progression^19,20^. Mice were mock-infected (vehicle alone), infected with 10^4^ PFU of WT virus alone, or with a mixture of WT virus and DVG-A TIPs. Approximately 10 DVG-A TIP genome copies per Zika virus infectious virion were used. As an additional control, mice received a mix of WT virus and supernatant from mock conditions of TIP production (“control TIPs,” generated in the absence of CPrME or NS1) (Fig. 5a). All mice lost weight by day 6, except mock-infected mice. At this time, TIP-treated (but not control TIP-treated) mice presented significantly lower weight loss than WT virus-infected mice (Fig. 5b). Further, unlike mice receiving WT virus alone or control treatment, TIP-treated mice presented significantly lower viremia at 2, 4, and 6 days p.i., with up to 2-log differences in virus titer (Fig. 5c). Viral loads in the footpads, spleens, ovaries, and brains were also 1–2 log lower in TIP-treated mice on day 6, underscoring the protective effect of this TIP (Fig. 5d). While DVG-A RNA in circulating blood was below detection levels, we confirmed the presence of DVG-A RNA in the footpads of TIP-treated mice, and at lower quantities in the spleens, ovaries, and brains (Fig. 5e). These results confirm that DVG-A TIPs successfully disseminate from the injection site to distal organs. Importantly, the safety profile of DVG-A TIPs was demonstrated in mice. In the absence of WT virus, DVG-A TIPs did not persist and did not disseminate to distal organs. The TIPs were degraded and undetectable within a day of administration (Supplementary Fig. 10).

In further support, we assessed TIP efficacy in a more immunologically competent mouse model^20^. C57BL/6 mice were treated with IFNAR1-blocking monoclonal antibody before infection with Zika virus, with or without TIPs. Weight loss was not monitored, as mice do not lose weight nor succumb to infection under these conditions^20^. Viremia was significantly lower in TIP-treated mice at 3 and 6 days p.i. (Fig. 5f). While we did not observe differences in viral loads in the footpads or spleens, viral loads in ovaries and brains were significantly lower in TIP-treated mice compared to control conditions, with up to 2-log difference in viral loads (Fig. 5g). As noted in AG129 mice, the high quantities of DVG-A at the injection site disseminated to the spleens, brains, and ovaries of TIP-treated mice (Fig. 5h).

### DVG-A impairs Zika virus dissemination and transmission in mosquitoes

**Fig. 6:**
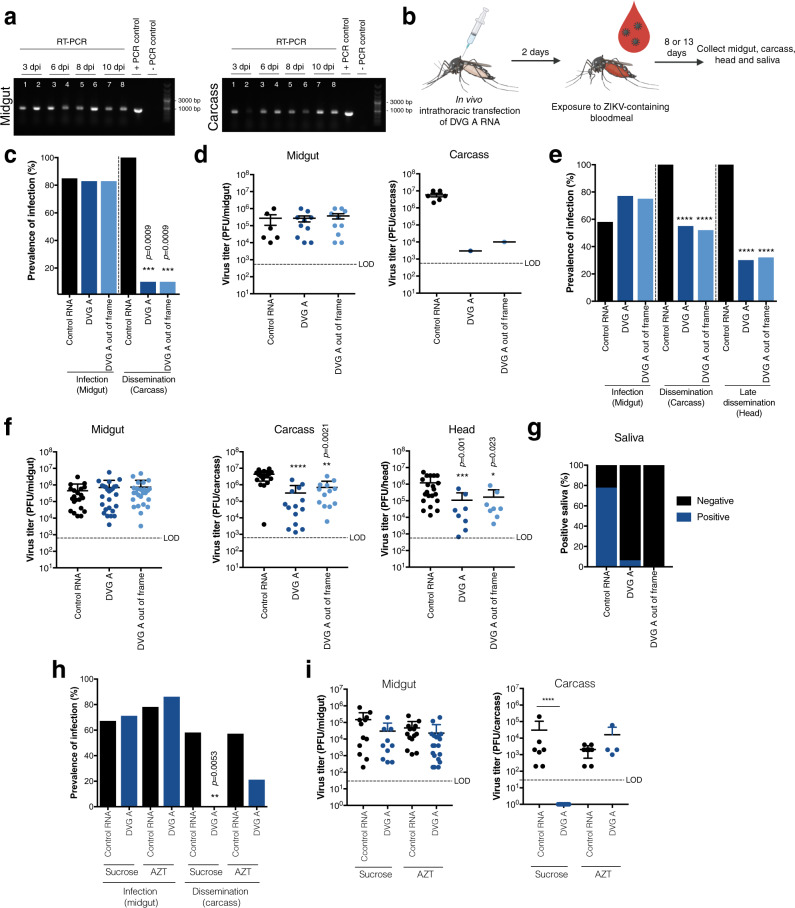
Zika virus DVG inhibits virus dissemination and blocks transmission in experimentally-infected mosquitoes. **a** Longevity of DVG-A in mosquitoes. *Ae. aegypti* mosquitoes were in vivo transfected with equimolar solutions of DVG-A or DVG-A out-of-frame RNA. At the indicated times post transfection, 5 mosquitoes were salivated and midgut, carcass, and head dissected. RNA was extracted from homogenates of a pool of the 5 mosquitoes and RT-PCR specific for the DVG was performed. Results from a representative experiment out of two is shown. Lanes 1, 3, 5, and 7 = DVG-A out-of-frame, Lanes 2, 4, 6, and 8 = DVG-A. Results for head and saliva samples are shown in Supplementary Fig. 11a. **b** Schematic representation of the experimental design. *Ae. aegypti* mosquitoes were injected with a transfection mix 0.02 pmoles of RNA. 2 days post-transfection, mosquitoes were fed a bloodmeal containing 2_x_10^6^ PFU/mL of Zika virus, and dissected at 8 or 13 d p.i. **c** Prevalence of Zika virus infection in the midgut or carcass of mosquitoes pre-transfected with control (*n* = 7), DVG-A (*n* = 12) or DVG-A out-of-frame (*n* = 12) RNA 8 d p.i. ***p = 0.0009 (by Fisher’s exact test, two-sided). **d** Viral load in infected mosquitoes from the midgut (control: *n* = 6; DVG-A: *n* = 10; DVG-A out-of-frame: *n* = 10) or carcass (control: *n* = 6; DVG-A: *n* = 1; DVG-A out-of-frame: *n* = 1) of the mosquitoes from (**c**). Mean and SD are shown. For midgut data, a one-way ANOVA with Dunnet’s multiple comparison was performed. For carcass, no statistical test was performed because due to *n* = 1. **e** Prevalence of Zika virus infection in the midgut, carcass or heads of mosquitoes pre-transfected with control (*n* = 36), DVG-A (*n* = 35) or DVG-A out-of-frame (*n* = 33) RNA 13 d p.i. ****p ≤ 0.0001 (by Fisher’s exact test, two-sided). **f** Viral load in the organs of the same infected mosquitoes from (**e**): midgut (control: *n* = 21; DVG-A: *n* = 26; DVG-A out-of-frame: *n* = 25), carcass (control: *n* = 21; DVG-A: *n* = 13; DVG-A out-of-frame: *n* = 13), or heads (control: *n* = 21; DVG-A: *n* = 8; DVG-A out-of-frame: *n* = 8). Mean and SD are shown. ****p ≤ 0.0001 (by one-way ANOVA with Dunnet’s multiple comparison). **g** Percentage Zika virus-positive saliva in mosquitoes pre-transfected with control (*n* = 9), DVG-A (*n* = 16) or DVG-A out-of-frame (*n* = 13) RNA 13 d p.i. **h** Prevalence of Zika virus infection in the midgut or carcass of sucrose- or AZT-fed mosquitoes that were injected with control or DVG-A RNA. Prevalence was assessed at 7 d p.i. Control RNA, sucrose fed (*n* = 18); DVG-A RNA, sucrose fed (*n* = 14); control RNA, AZT fed (*n* = 18); DVG-A RNA, AZT fed (*n* = 22). **p = 0.0053 (by Fisher’s exact test, two-sided). **i** Viral load in the midgut (control, sucrose fed: *n* = 12; DVG-A, sucrose fed: *n* = 10; control, AZT fed: *n* = 14; DVG-A, sucrose fed: *n* = 19) or carcass (control: *n* = 7; DVG-A: *n* = 10<LOD; control, AZT fed: *n* = 8; DVG-A, sucrose fed: *n* = 4) of the same mosquitoes from (**h**). Mean and SD are shown. ****p ≤ 0.0001 (by one-way ANOVA with Dunnet’s multiple comparison). Data from two independent experiments is shown. LOD = limit of detection. Control RNA: pTRI-Xef RNA. Source data are provided as a Source Data file.

Finally, we investigated DVG-A inhibitory capacity in the mosquito vector. First, *Ae. aegypti* mosquitoes were injected with a mix containing equimolar amounts of RNA (DVG-A, DVG-A_out-of-frame or control RNA), anatomical compartments were dissected, and the longevity of DVGs in vivo was determined by RT-PCR. DVG-A and DVG-A_out-of-frame RNA was detectable in the midgut and carcass of transfected mosquitoes for at least 10 days post transfection, even in the absence of virus (Fig. 6a), while no RNA could be detected in head or saliva samples (Supplementary Fig. 11a).

Next, we fed mosquitoes an infectious blood meal containing Zika virus two days after injection of DVG or control RNA, and examined viral loads in mosquito compartments 8 days after infection (Fig. 6b). The midguts and carcasses of mosquitoes were scored for the presence of virus to determine the infection and dissemination rates, respectively. Similar infection rates were observed, regardless of the pre-treatment mosquitoes received. While virus disseminated in 100% of mosquitoes that underwent control RNA pre-treatment, only 10% of mosquitoes that received either version of DVG-A had disseminated virus (Fig. 6c). In both cases, the viral titer in the single positive mosquito carcass from each group was 2-3-logs lower than the mean viral load in control RNA-transfected mosquito carcasses, whereas no differences were found in midgut viral loads (Fig. 6d).

To determine if dissemination was either completely blocked or delayed by DVG-A treatment, we repeated the experiment at a later time point (13 days post oral exposure to Zika virus). DVG-A RNA was still present in mosquitoes at this time point (Supplementary Fig. 11b). It should be noted that a lower percentage of mosquitoes were productively infected compared to the previous experiment (58%, 77%, and 75% infection rates for control, DVG-A, or DVG-A_out-of-frame treated mosquitoes, respectively) (Fig. 6e). As in the previous experiment, 100% of control RNA-treated mosquitoes presented disseminated virus, whereas virus had disseminated in only 55% and 52% of DVG-A or DVG-A_out-of-frame treated mosquitoes (Fig. 6e). While 100% of heads (a proxy for late dissemination) of control RNA-treated mosquitoes were also virus-positive, only 30% or 32% DVG-A or DVG-A out-of-frame treated mosquitoes manifested late virus dissemination (Fig. 6e). Further, there were no differences in viral load in the midgut, but the viral load was significantly lower in the carcass and heads of both DVG-treated groups than in control mosquitoes (Fig. 6f). Thus, both DVG-A and DVG-A_out-of-frame block or significantly delay Zika virus dissemination, and for mosquitoes in which dissemination is not blocked, disseminated viral loads are significantly lower than in control mosquitoes.

These observations prompted us to question if lower amounts of disseminated virus could result in impaired transmission. We thus analyzed saliva that was collected from a subset of mosquitoes in the last experiment. While infectious virus was present in the saliva of 78% of carcass-positive mosquitoes that received control RNA treatment (*n* = 9), only one carcass-positive mosquito (6.25%) contained infectious virus for DVG-A treated mosquitoes (*n* = 16), and no saliva of DVG-A_out-of-frame treated mosquitoes (*n* = 13) had infectious virus (Fig. 6g).

We have previously shown that arboviruses establish persistent infections in the mosquito host via the production of a viral-derived DNA (vDNA) and that DVGs can serve as templates for the synthesis of this vDNA^21,22^. To assess whether the Zika virus DVG-induced protection in mosquitoes was mediated by the presence of vDNA, DVG-A, or control RNA-injected mosquitoes were treated with azidothymidine (AZT), a compound that inhibits reverse transcription and thus the synthesis of the vDNA^22,23^. AZT treatment rescued dissemination rates (Fig. 6h) and viral load of disseminated virus (Fig. 6i) at 7 days post infection, confirming that in the mosquito host, Zika virus DVGs mediate an RNAi-dependent inhibition of WT virus via vDNA production.

## Discussion

The potential to use DVGs as antivirals or prophylactics has previously been proposed in studies showing that virus stocks rich in DVGs can interfere with disease outcomes in mice or ferrets^24–27^. To date, synthetically engineered DVGs with interfering potential were shown to be effective as prophylactic (vaccine adjuvants) or therapeutic antivirals for two respiratory viruses, RSV and influenza A, in mouse models^28–30^. DVG diversity has been largely overlooked in the field, with only few DVGs per virus family described. This is because early reports relied on classic methods of isolation, such as RT-PCR amplification, that bias toward the shortest, most readily amplified, and abundant DVGs. Here, using a combined approach based on experimental evolution, NGS, and computational methods to measure fitness, we explored the vast DVG sequence space in three different flaviviruses to identify a top candidate DVG with potential to interfere with WT virus infection. Indeed our NGS data show that deletions occur randomly throughout the viral genome with no apparent bias (Supplementary Fig. 6a–c) and suggest that the subset observed at higher frequency is dictated by biological constraints, such as the retention of coding sequences, replication elements, and packaging signals. Our results demonstrate that DVG-A meets all these conditions, and could explain why the DVG-A cluster exists.

One key element to success was relying on high replicate numbers in the passage series that is customary in evolutionary sciences, rather than the minimal triplicate that is the default in molecular biology. Triplicate passage simply would not have been enough to observe convergent evolution in experiments, as the variation among replicates illustrated (Supplementary Fig. 4). The methodology described here to identify DVG therapeutic candidates could be applied to any virus species. While we focused solely on deletions, other types of DVGs such as rearrangements, insertions or a combination of these that cannot be easily characterized by short-read sequencing may also constitute therapeutic candidates. Emerging sequencing technologies such as long-read sequencing or ClickSeq^31^ and analysis tools such as ViReMa^32^ will allow us to identify more complex genomes in a high-throughput manner.

As expected, our list included DVGs that resemble those identified by more classic methods, similar to the dengue virus DVGs with large deletions containing only key regulatory elements at the ends of the genome^33^. However, our analysis predicted and we experimentally confirmed that such DVGs did not have sustained fitness and showed no antiviral activity. This observation questions the classical notion that competition between DVGs and WT genomes is mostly a matter of genome size, where the smallest DVGs would be replicated at much faster rates and outcompete WT virus. Instead, we found that a DVG with a deletion of the structural proteins, that retains non-structural protein sequences except NS1, was most fit. This same DVG was identified as high fitness in other flaviviruses and found to be most capable of inhibiting WT virus. DVGs similar to DVG-A have been reported in flavivirus persistent infections in vitro and in natural isolates^7,34–38^, although their naturally occurring frequency was seemingly not high enough to successfully interfere with WT virus.

By packaging the fittest DVG candidate into VLPs, we show that these TIPs are not only active at the site of injection in the mammalian host, but disseminate to distal organs that are primary targets of Zika virus infection, an important feature for combatting most arbovirus infections. Importantly, the reduced viral loads in brains and ovaries of TIP-treated mice suggest that DVGs may help avert the neurological complications of Zika virus infection in severe cases of the disease^39,40^, or vertical and/or sexual transmission of the virus^41–45^. Thus, our results provide a proof of concept that DVGs can target infection against flaviviruses such as Zika virus in the vertebrate acute infection cycle. A limitation of our study, in terms of antiviral efficacy, is that we did not evaluate to what extent TIPs can be administered after virus exposure, since we only administered TIPs at the same time as virus infection. The success of this DVG in the vertebrate host may lie in its ability to compete and interfere with WT on more than one level. First, it competes with WT virus in the most classic parasitic sense by ‘stealing’ key viral proteins away from virus, which it requires for genome replication and packaging (e.g. NS1 or structural proteins). Second, while it may interfere with WT replication by taking viral replicase proteins away from the virus, it may not solely rely on WT-derived non-structural proteins. In fact, while DVG-A is not a true replicon, and it behaves like an inert replicon with an ‘On’ switch that only becomes active once NS1 is supplied in *trans*, whereby it then produces its own non-structural proteins. These DVG-encoded proteins can then enter into competition with WT virus proteins for cellular resources as well.

While studies on DVGs from other viruses showed them to be strong triggers of innate immune responses affecting viral replication^3,14,46–50^, DVG-A did not upregulate these interferon-related genes. Further, the mouse models used here are either deficient in interferon α/β and γ receptors or have temporally diminished IFN α/β signaling. Thus, the protection conferred by DVG-A in these mice provides further evidence that the mechanism of action by DVG-A is independent of the type I and II interferon responses. Presumably, DVGs such as DVG-A with internal deletions are weaker activators of the interferon response than those used in previous studies, such as copy-back DVGs, which can form dsRNA known to be a critical factor in immunostimulation^46^.

Contrary to what was observed for acute infections in the vertebrate host, DVG-A was antiviral in mosquito cells regardless of whether the ORF was maintained, suggesting that the antiviral activity of the DVG is independent of the proteins or functions it encodes. Indeed, RNAi is the major antiviral pathway in insects, and our work shows that inhibition by DVG-A relies on an intact RNAi pathway. Furthermore, we show that the major mechanism by which DVGs inhibit viral infection in insects is through the generation of a DNA form of the DVG RNA which boosts the RNAi response. Indeed, our previous work suggested that DVGs are a preferential template from which to for the viral DNA form^21^. Our results provide a proof of concept that DVGs could constitute a new vector control strategy for any arbovirus. Previous work showed that West Nile virus stocks containing DVGs correlated with decreased infection rates in *Culex* mosquitoes^7^. Here, we show that synthetic DVG-A RNA introduced into mosquitoes can block virus dissemination in mosquito bodies by 50% and most importantly, reduce transmission by 90%. Since inhibition occurred regardless of the retention of the reading frame, its plausible that a large array of DVGs presenting random deletions could be as effective at reducing transmission in mosquitoes if expressed at high enough amounts. Given the recent advances in mosquito genome editing techniques^51–53^, it would be interesting to assess the use of knock-in mosquitoes carrying DVGs or DVG-derived sequences in endemic settings and to apply this approach to other arboviruses to reduce global disease burden.

Arboviral diseases constitute a public health threat worldwide. To tackle the (re-)emergence of arboviruses, novel strategies for therapeutic and prophylactic treatments, as well as vector control are required. Our study provides an in-depth approach to identify fit, competitive DVG molecules naturally generated by a virus and most suitable for interfering with infection, and underscores the potential of DVGs to address these needs; not only as therapeutics by reducing virulence in the vertebrate host, but also as a vector control by limiting virus dissemination and transmission during persistent infection of the natural mosquito vector. Given that virtually all RNA viruses can generate DVGs, our experimental and computational method to select high fitness DVG may be useful as a global intervention strategy against RNA viruses.

## Methods

### Cells and viruses

Vero and Vero-E6 cells were maintained in Dulbecco’s modified Eagle’s medium (DMEM) containing 10% (v/v) fetal bovine serum (FBS, Invitrogen). HEK-293T cells were maintained in the same medium supplemented with 1% (v/v) non-essential amino acids. SW-13 cells were maintained in Leibovitz’s L-15 medium (Life Technologies) with 2mM L-glutamine and 10% (v/v) FBS. C6/36 and U4.4 were maintained in Leibovitz’s L-15 medium (Life Technologies) supplemented with 10% FBS (v/v), 1% (v/v) non-essential amino acids and 1% (v/v) tryptose phosphate broth (Sigma). All cell lines were supplemented with 5 units/mL penicillin and 5 μg/mL streptomycin (Life Technologies). Mammalian cell lines were maintained in a humidified atmosphere at 37 °C with 5% CO_2_. C6/36 and U4.4 cells were maintained at 28 °C without CO_2_.

The Zika virus strain used in this study is the prototype African MR-766 strain, derived from a previously described infectious clone^54^. Rescue of WT Zika virus was carried out by transfection of the infectious clone in HEK-293T cells (TransIT-LT1, Mirus Bio) according to the manufacturer’s instructions. 3-4 days post-transfection (p.t.), the cell culture supernatant was clarified by centrifugation. Virus stocks were then prepared in Vero cells, by infecting cells with a multiplicity of infection (MOI) of 0.01 plaque-forming units (PFU)/cell. 5-6 days post-infection (p.i.) the cell culture supernatant was clarified by centrifugation, and virus titers determined by plaque assay in Vero-E6 cells.

The yellow fever virus 17D strain was derived from pACNR/FLYF plasmid containing the full-length infectious YF17D genome under a SP6 promoter^55^.

West Nile virus (Israeli strain IS-98) was generated from the “two-plasmid” system infectious clone previously described^56^.

### Serial passaging of Zika virus to generate virus populations enriched in DVGs

Zika virus stocks were used to inoculate Vero or C6/36 cells at a low (0.01 PFU/cell) or high (20 PFU/cell) MOI in 12-well plates. 3 or 5 days p.i. (for Vero or C6/36 cells, respectively) the cell culture supernatant was clarified by centrifugation and used for the next passage. After passage 1 (p1), blind passaging was performed, using 5 μl or 300 μl of the virus from the previous passage per well for low and high MOI conditions, respectively. All samples were titrated and we confirmed that indeed the high and low MOI conditions were respected (Supplementary Fig. 1). Yellow fever virus was passaged at high (5 FFU/cell) and low (0.1 FFU/cell) MOI in SW13 cells for 2 days. West Nile virus was passaged similarly, in C6/36 cells.

### Viral RNA sequencing

RNA from clarified cell culture supernatants was extracted using TRI reagent (Sigma). Purified viral RNA was quantified with a Qubit fluorometer (Invitrogen) and used for sequencing by next-generation RNA sequencing. Libraries were prepared using the NEBNext Ultra II RNA Library preparation kit for Illumina (New England Biolabs) and loaded in a NextSeq500/550 Mid output kit v2.5 (Illumina), for sequencing in a NextSeq500 (151 cycles, 8 nucleotides of index).

### Sequence data processing and analysis

Reads were first demultiplexed using the bcl2fastq conversion software (Illumina). Next, the reads were adapter- and quality-trimmed using BBDuk and optical duplicates removed using Clumpify. Both BBDuk and Clumpify are part of the BBTools and BBMap suite version 38.06 (https://sourceforge.net/projects/bbmap/). Reads were then aligned to the reference genome using BBMap and deletions called with the BBMap variant caller CallVariants. The number of reads mapping to a deletion was normalized relative to the total number of reads that aligned to the reference genome. This value was multiplied by 10^6^ to obtain deletion event reads per million reads (RPM), as described previously^2^.

### Computational determination of neighborhoods enriched with deletions

“Junction” reads (reads that align to the reference virus genome but not as a continuous alignment) were grouped into clusters of fragments with similar start and end deletion positions. Specifically, deleted fragments with less than 10 positions in divergence in their start and end positions were grouped in a cluster (“elementary deletion cluster”). The size of the elementary deletion cluster is a “junction coverage” of the deletions’ narrow start and end position interval by reads with the corresponding junctions. The ratio of “junction coverage” to the sum of “junction coverage” and “continuous coverage” (derived from reads with continuous alignment that cover these deletions’ start and end intervals) is a frequency of the elementary deletion cluster.

The most abundant deletions (with frequencies higher than 0.0085%) from all high MOI replicates were selected to determine regions in the viral genome in which deletions were more predominant. Since deletions with this threshold are not uniformly distributed, enriched areas can be found unequivocally by the applied nested neighborhood algorithm in a 2-dimensional plane composed of start and end positions of deletions as X and Y coordinates. This method allows detecting the area (neighborhood) enriched by points around a certain center by sequentially extending the neighborhood’s border (distance to the center from the next closest point) and calculating the neighborhood’s fractal dimension to get more accurate *p* values of enrichments on each step. For methods describing the calculation of fractal dimension, refer to the “Fractal dimensions of neighborhoods” section.

Putative centers of the neighborhoods enriched by deletions were detected on the plane using a grid method. Namely, a number of deletions (“points”) were randomly selected as the grid references. For all points, distances to these reference points were calculated. For every point, a product of all-rounded logs of its Euclidean distances to the reference points was used as a hashing index. The hashing indexes of all points were sorted, and big enough islands of points (threshold ≥ 7 elementary deletion points) in the sorting that have the same hash index were considered as containing putative centers of significant enrichment. Any point of the island can be used as a center for the subsequent determination of the center’s neighborhood most significantly enriched by points/deletions. Let the null hypothesis assumption be that all points/deletions are uniformly distributed on the start/end plane (i.e., no enrichments assumption). Then probability for a number of points to be in a neighborhood of radius *r* from the center with volume (or an area if the space is a two-dimensional plane) *V*_*r*_ can be calculated from Poisson distribution. Indeed, if points are uniformly distributed in the neighborhood with radius *R*, *R* > *r* of volume *V*_*R*_., then the number of points in a neighborhood with the same center and radius *r* (therefore, with volume *V*_*r*_) will be a random variable, having a Poisson distribution with the parameter $\lambda = \alpha \cdot r^k$, where *k* is the dimension of the space, and *α* is the density of the uniform distribution of *n* points in *V*_*R*_ $\left( {\alpha = \frac{n}{{R^k}}} \right)$. Thus, *n* is proportional to *Vr*. The probability (*P*) of finding more or equal to *m* points in a neighborhood with radius *r* (pvalue) will be:

1 $$P_m\left( r \right) = \mathop {\sum}\nolimits_{i = m}^\infty {\frac{{\lambda ^i}}{{i!}}e^{ - \lambda } = \mathop {\sum}\nolimits_{i = m}^\infty {\frac{{\left( {\alpha \cdot r^k} \right)^i}}{{i!}}e^{ - \left( {\alpha \cdot r^k} \right)}} }$$

All deletions/points were sorted according to closeness to the selected central point. Those regions that are most enriched by points were determined as follows. In the sorting, let us consider a transition from a neighborhood with radius *r*_*t*_, which is equal to a distance from the center to point *t* in the sorting, to a neighborhood with radius $r_{t + 1}$. The Poisson *p* value of the enrichment of the neighborhood of radius *r*_*t*_ containing *t* points is calculated from the perspective of the extended neighborhood with the radius $r_{t + 1}$ and assumptions that its *t* + 1 points are uniformly distributed in this volume of space of fractal dimension: the fractal dimension is defined by the sequence of distances from the *t* + 1 points to the center. For non-uniform dense areas, the Hausdorff fractal dimension is higher than the geometrical dimension of the plane equal to two. This higher dimension makes a drop of *p* value sharper on a transition from *t* to *t* + 1 than in two-dimensional space. The *t*-neighborhood with the most significant Poisson *p* value was selected as the best neighborhood, i.e., the one that is most enriched by deletions/points.

### Statistical significance of deletion frequency within enriched neighborhoods

We assumed a binomial distribution for a number of “junction coverage” events in trials numbered as the sum of numbers of all “junction coverage” and all “continuous coverage” events. Since the “continuous coverage” was high, the 95% confidence interval for the “junction coverage” frequency was evaluated via a chi-square distribution for binomial log-likelihood ratio (Wilks theorem). In this way, the significance of the deletion, i.e., its deviation from zero, can be measured in standard deviation (SD) units of the normal distribution (z-score of the elementary deletion). This SD-unit measurement is based on the consideration that the 95% confidence interval of the normal distribution nearly equals to four SDs.

Significance of a cluster of deletions in a neighborhood was inferred by calculating the z-score of the neighborhood. For each passage, the neighborhood z-score was calculated as the sum of z-scores of its deletions divided by the square root of the total number of deletions in the neighborhood. Since significance of each deletion is measured in SD and therefore distributed normally N(0,1), the calculated z-score for total significance of all deletions in the neighborhood is also distributed normally N(0,1) and therefore easily translated to a *p* value. Thus, the dynamics of the neighborhood z-scores across passages show how the total significance of the neighborhood’s set of deletion frequencies increases or decreases.

### Fractal dimensions of neighborhoods

Fractal dimensions of neighborhoods were calculated for a more sensitive determination of the most enriched neighborhoods. A fractal dimension for a neighborhood of a center of radius $r_{t + 1}$ with *t* + 1 closest distinct points in it can be calculated as follows. Let each point in the neighborhood be exchanged with the same neighborhood compressed with the $\frac{{minimumDistance}}{{maximumDistance}} \cong \frac{{r_1}}{{2 \cdot r_{t + 1}}}$ ratio. Next, each point in the compressed neighborhood *N*_1_ will be exchanged with the double compressed original neighborhood: compressed with the $\left( {\frac{{r_1}}{{2 \cdot r_{t + 1}}}} \right)^2$ ratio, next each point of the double compressed neighborhood will be exchanged with the triple compressed neighborhood: $\left( {\frac{{r_1}}{{2 \cdot r_{t + 1}}}} \right)^3$ ratio, and so on.

In this nested construction, balls of diminishing radiuses cover the infinite sequence of the nested compressed self-similar (fractal) ball-neighborhoods. Hence, the Hausdorff fractal dimension of the ball-neighborhood can be calculated as a minimal dimension *d* of a ball (volume without compression is $r_{t + 1}^d$) in order to get the lowest estimation for sum of volumes of infinite series of all diminishing balls that cover all points in this nested fractal construction.

The Hausdorff content of this series is

2 $$H^d = \mathop {{{\mathrm{lim}}}}\limits_{n \to \infty } \left\{ {\left( {t + 1} \right)^n \cdot \left[ {r_{t + 1} \cdot \left( {\frac{{r_1}}{{2 \cdot r_{t + 1}}}} \right)^n} \right]^d} \right\}$$

Dimension *d* of this Hausdorff content of the ball-neighborhood is defined as:

3 $$dim\left( {H^d} \right) = inf\left\langle {d\, > \, 0:\mathop {{{\mathrm{lim}}}}\limits_{n \to \infty } \left\{ {\left( {t + 1} \right)^n \cdot \left[ {r_{t + 1} \cdot \left( {\frac{{r_1}}{{2 \cdot r_{t + 1}}}} \right)^n} \right]^d} \right\} = 0} \right\rangle$$

Alternatively, the equivalent formula will be as follows:

4 $$dim\left( {H^d} \right) = inf\left\langle {d\, > \, 0:\mathop {{{\mathrm{lim}}}}\limits_{n \to \infty } \left\{ {\left( {r_{t + 1}} \right)^d \cdot \left[ {\left( {t + 1} \right) \cdot \left( {\frac{{r_1}}{{2 \cdot r_{t + 1}}}} \right)^d} \right]^n} \right\} = 0} \right\rangle$$

This infimum for dimension is calculated as:

5 $$d = \frac{{{\mathrm{ln}}\left( {t + 1} \right)}}{{\ln \left( {2r_{t + 1}} \right) - {\mathrm{ln}}\left( {r_1} \right)}}$$

Indeed, for the above limit under $n \to \infty$ to be equal to zero the inequality as follows has to be true:

6 $$\left( {t + 1} \right) \cdot \left( {\frac{{r_1}}{{2 \cdot r_{t + 1}}}} \right)^d < 1$$

Taking the logarithm from both parts, the inequality will be transformed into equivalent:

7 $$ln\left( {t + 1} \right) + d \cdot ln\left( {\frac{{r_1}}{{2 \cdot r_{t + 1}}}} \right) < 0$$

8 $$d > \frac{{ln\left( {t + 1} \right)}}{{ - ln\left( {\frac{{r_1}}{{2 \cdot r_{t + 1}}}} \right)}}$$

Taking infimum we will get:

9 $$d = \frac{{ln\left( {t + 1} \right)}}{{\ln \left( {2 \cdot r_{t + 1}} \right) - {\mathrm{ln}}\left( {r_1} \right)}}$$

### Generation of DVG clones

The Zika virus infectious clone was used as a template for the generation of DVG-encoding clones, using primers to excise deleted region of interest. The deleted nucleotides in each DVG clone were: 581-3250 for DVG-A, 339-9033 for DVG-B, and 5351-5668 for DVG-C. Zika virus replicons were generated as previously described^12^. Briefly, the region encoding the structural proteins (C, Pr, M, and E) was replaced by a cassette containing C38-NanoLuc-2A-E30 (the N-terminal 38 amino acids of C protein, NanoLuc reporter, foot-and-mouth disease virus 2 A protease, and the C-terminal 30 amino acids of the E protein). The N-terminal 38 amino acids of the C protein contains the *cis*-acting element of nucleotides complementary to the 3’ cyclization sequence^57^. The C-terminal 30 amino acids of E correspond to the transmembrane domain required to maintain the topology of NS1 in the ER. In addition, the intron present in NS1 in the Zika virus clone was removed. A mutant replicon plasmid was generated for use as a negative control, by replacing the catalytic motif G_664_D_665_D_666_ in the viral polymerase NS5 by three alanine residues (inactive replicon). The DVG-A reporter clone was generated similarly; with NanoLuc-2A introduced at the deletion site, namely between the N-terminal 38 amino acids of Pr and the C-terminal 98 amino acids of NS1.

### Replicon assays

To generate replicon RNA, the full replicon cassette from CMV-driven clones was amplified using primers annealing to the backbone of the plasmid, and in which the forward primer contained an SP6 sequence. RNA was in vitro transcribed from the PCR product (mMESSAGE mMACHINE SP6 Transcription kit, Thermo Fisher), and purified using the RNeasy mini kit (QIAGEN). RNA was quantified using a Qubit fluorometer (Invitrogen) and used for transfection of HEK-293T or Vero cells. Briefly, sub-confluent cells in 24-well plates were transfected with 100 ng of replicon RNA using TransIT-mRNA transfection kit (Mirus Bio). Over a 72 h time course, the supernatant was removed and cells lysed in passive lysis buffer (Promega). Luciferase expression was measured using the Nano-Glo luciferase assay system (Promega) in a Tecan infinite M200 pro plate reader (Tecan group).

Assays in which the effect of non-structural proteins on DVG replicon activity was studied were performed as described above, except HEK-293T cells were transfected with E_30_-NS, E_30_-NS_*NS1_, E_30_-NS_*NS2A_, E_30_-NS1 plasmids using LT-1 transfection reagent (Mirus Bio) 24 h prior to reporter RNA transfection. E_30_-NS is a CMV-driven clone encoding the Zika virus C-terminal 30 amino acids of E and all nonstructural proteins. E_30_-NS_*NS1_, E_30_-NS_*NS2A_ plasmids are identical to E_30_-NS, except a stop codon introduced as the first amino acid of NS1 or NS2A, respectively. E_30_-NS1 encodes only E_30_ and NS1. We performed these assays in HEK-293T cells because of their high transfection efficiency, which proved to be important for the successful transfection of both DNA and RNA molecules. To confirm successful expression from the clones, we generated a C-terminal, V5-tagged version of E_30_-NS1. Cell lysates of transfected cells were collected at 24 h p.t. in RIPA buffer (Sigma). V5-tagged NS1 was detected by conventional western blotting, using an anti-V5 antibody (Abcam; ab27671) and anti-mouse HRP linked antibody (GE Healthcare Life Sciences).

### DVG inhibitory activity in vitro

HEK-293T cells were transfected with the DVG-encoding clones along with the WT Zika virus clone at different molar ratios (1:10, 1:1 and 10:1 DVG:WT) using LT-1 transfection reagent (Mirus Bio). As controls, additional WT virus clone or a GFP-encoding clone (control clone unrelated to Zika virus) were used. 72 h p.t. WT virus titers were determined by plaque assay.

Due to the low efficacy of the CMV promoter in invertebrate cells^58^, we tested DVG-induced inhibition in mosquito cells by transfection of DVG RNA. Briefly, PCR amplicons of the full DVG sequences were generated using primers with an SP6 promoter at the 5’ end. SP6 in vitro transcription was then performed using the PCR product as a template (mMessage mMachine SP6 Transcription kit, ThermoFisher). Following DNase treatment, the reaction was cleaned up (RNeasy MinElute Cleanup kit, QIAGEN) and RNA quantified with a Qubit fluorimeter (Invitrogen). 0.062, 0.125, or 0.25 pmoles DVG or pTRI Xef control RNA were transfected into sub-confluent C6/36 or U4.4 cells seeded in a 48-well plate (Lipofectamine LTX, ThermoFisher). The next day, cells were infected with Zika virus at an MOI 0.1 PFU/cell and the medium replaced. 5 days p.i., infectious virus in the cell culture medium was quantified by plaque assay. For assessing the effect of DVG RNA in Ago-2 knockdown cells, co-transfection of the DVG or control RNA was carried out with Ago-2 or control dsRNA.

### Induction of antiviral genes

For quantitative reverse transcription PCR (RT-qPCR) of antiviral genes, total cellular RNA was reverse transcribed using the Maxima H Minus First Strand cDNA Synthesis Kit with random primers (ThermoFisher). cDNA was used for qPCR using specific primers (Supplementary Table 3) and the Power SYBR Green PCR Master Mixture (Applied Biosystems) on a Step-One-Plus Real-Time PCR thermocycler (Applied Biosystems). The relative fold gene expression of genes was calculated relative to GAPDH using the delta-delta Ct method, comparing to mock conditions.

### Knockdown of Ago-2 in U4.4 cells

RNA from U4.4 cells was extracted (TRIzol, Thermo Fisher) and cDNA generated using the Maxima First Strand cDNA Synthesis Kit with random primers (Thermo Fisher). A 686 bp Ago-2 region was amplified from the cDNA using specific T7-promoter flanked primers (T7-Ago2-F and T7-Ago2-R, Supplementary Table 3). PCR products were cleaned up (NucleoSpin Gel and PCR Clean-up, Macherey Nagel) and used for dsRNA production (MEGAScript RNAi kit, Thermo Fisher). Knock-down of Ago-2 was performed by transfection of 250 ng dsRNA per well in sub-confluent U4.4 cells seeded in a 48 well plate (Lipofectamine LTX, Thermo Fisher). Control knock-downs were carried out using the control dsRNA provided in the MEGAScript RNAi kit.

The efficiency of Ago-2 knockdown was assessed by mRNA quantification. Briefly, 24 h following dsRNA transfection, cells were lysed in RNA lysis buffer and RNA extracted using the *Quick*-RNA 96 kit (Zymo), including the in-column DNAse digestion step to remove genomic DNA. RT-qPCR was carried out using the Luna Universal One-Step RT-qPCR kit (New England Biolabs) following the manufacturer’s instructions, with Ago-2 and actin specific primers (Supplementary Table 3), on a Step-One-Plus Real-Time PCR thermocycler (Applied Biosystems). Quantification of Ago-2 mRNA expression was calculated relative to actin mRNA using the delta-delta Ct method.

### Determination of DVG packaging capacity

Uninfected or infected Vero cells (producer cells, MOI 1 PFU/cell at 24 h) in 24-well plates were transfected with 100 ng of DVG replicon RNA, WT replicon (positive control), or inactive replicon (negative control), using TransIT-mRNA transfection reagent (Mirus Bio). 48 h p.t. the supernatant was endonuclease-treated (25 Units/μl BaseMuncher, Expedeon) and used to infect naïve Vero cells in 24-well plates (recipient cells). Replicon activity in recipient cells was assessed 24 h p.i. Donor and recipient cells were lysed in passive lysis buffer (Promega) and luciferase activity measured using the Nano-Glo luciferase assay system (Promega) in a Tecan infinite M200 pro plate reader (Tecan group).

### Generation of DVG-containing VLPs

DVG-containing VLPs were generated as previously described^13^. Briefly, HEK-293T cells seeded in a 24 well plate were transfected with a mix of 200 ng of a CPrME-encoding plasmid, 200 ng of E_30_-NS1, and 200 ng of the DVG-encoding plasmid (LT-1 transfection reagent, Mirus Bio). 72 h p.t., the medium was clarified by centrifugation. Naïve recipient cells were infected with producer cell supernatant following endonuclease (Basemuncher, Expedeon) treatment, and successful packaging determined with luciferase measurement when using reporter DVG clones, or by RT-qPCR when using native DVG.

### RT-qPCR for WT or DVG RNA

Detection of WT and DVG RNA was performed by RT-qPCR, using WT and DVG specific primers and probes. For WT RNA, the probe and primers were designed to bind to the deleted region in the DVG. DVG-specific primers were designed such that a 113 nt region containing the deleted part of the genome is amplified by primers binding to the Pr gene and the NS1 gene. Amplification of this region in WT virus RNA would result in a significantly larger product (2783 nt). In addition, the DVG recognizing probe was designed such that it would bind to the breakpoint of the deletion. The primer sequences are shown in Supplementary Table 3. DVG or WT RT-qPCR was carried out using the TaqMan RNA-to-Ct One-step RT-PCR kit (Applied Biosystems) or the Luna Universal One-Step RT-qPCR kit (Abcam) on a Step-One-Plus Real-Time PCR thermocycler (Applied Biosystems). The number of RNA genomes was derived from a standard curve produced in each RT-qPCR run using in vitro generated DVG or WT RNA.

### Mouse experiments

AG129 mice^59^ were bred in a specific-pathogen-free facility at Institut Pasteur’s animal facility, and C57BL/6 mice were obtained from Charles River Laboratories. Infected adult mice were housed in BSL-3 level isolators and handled in compliance with the Animal Committee regulations and guidelines of Institut Pasteur Paris France, under the 2010/63 European Union Council directive. Animal protocols were approved by the Ethics Committee on Animal Experimentation (CETEA) under dossier number dap160116/CHCST 18.176 and the USAMRMC Animal Care and Use Review Office (ACURO), under the protocol number DARPA-5417.04. Mice were monitored daily, with food and water supplied ad libitum. Endpoints were defined and mice humanely euthanized when these were reached.

4-6 week old AG129 or C57BL/6 female mice were given a mix of Ketamine(10 mg/mL)/Xylazine (1 mg/mL) by intraperitoneal injection. Once anesthetized, mice were inoculated by a subcutaneous (footpad) route with vehicle (DMEM media), 10^4^ PFU of Zika virus alone, or complemented with DVG-containing VLPs (TIPs). C57BL/6 mice were treated with 2 mg of an IFNAR1 blocking mouse MAb (MAR-5A3, Euromedex) by intraperitoneal injection one day prior to infection. Weight loss and viremia were monitored at the indicated times after infection. Blood was collected from the facial vein in Microtainer blood collection tubes (BD) and allowed to clot at room temperature. Serum was separated by centrifugation and stored at −80 °C. Viremia was quantified either by plaque assay or RT-qPCR on unextracted and diluted serum samples, as described previously^60^. Infected mice were euthanized by cervical dislocation. Spleen, ovaries, brain, and injected footpads were harvested and homogenized with 600 μl DMEM supplemented with 2% FBS in Precellys tubes containing ceramic beads, using a Precellys 24 homogenizer (Bertin Technologies) at 5000 rpm for 2 cycles of 20 seconds. Homogenates were cleared by centrifugation, and the supernatant stored at −80 °C until virus titration or RT-qPCR. Viral burden in organs was measured either by plaque assay (expressed as PFU/g for organs) or by RT-qPCR (expressed as PFU equivalents relative to GAPDH) following RNA extraction using Direct-zol-96 (Zymo). The number of DVG or WT RNA genomes in mouse organs was normalized to GAPDH genomes, derived from a standard curve using mouse GAPDH primers (Supplementary Table 3) and RNA extracted from the respective organ of uninfected mice.

### Mosquito experiments

6-8 days old female *Ae. aegypti* mosquitoes (1 colony, F7 generation, collected originally in Kamphaeng Phet Province, Thailand) were cold-anesthetized and injected with a transfection mix of CellFectin II reagent (Thermo Fisher) containing Liebovitz’s L-15 medium, 0.02 pmoles of pTRI Xef control or DVG RNA in a total volume of 200 nL. The injection was performed intra-thoracically using a nanoinjector (Nanoject III, Drummond Scientific) and a glass capillary needle. 2 days after transfection and following starvation for one day, mosquitoes were allowed to feed for 30 min on an artificial blood meal at 37 °C containing previously washed human blood (2 mL), Zika virus (1 mL, 6×10^6^ PFU) and 5 mM ATP. Engorged mosquitoes were counted, selected and maintained at 28 °C in a humidified atmosphere (70%), supplemented with 10% sucrose *ad libitum*. For experiments where mosquitoes were fed with azidothymidine (AZT), mosquitoes were supplemented daily with sucrose-containing 37 mM AZT (Adenosine 5′-triphosphate disodium salt solution A6559, Sigma-Aldrich). 8 days after the blood meal, mosquitoes were cold-anesthetized and dissected. Midguts and carcasses were collected in microtubes containing steel beads (5 mm diameter) and 200 μL DMEM supplemented with 2% FBS. 13 days after the blood meal, mosquitoes were salivated in a tip containing 5 μL FBS, followed by the collection of heads, midgut, and carcass. Body parts were homogenized using a TissueLyser II (QIAGEN) at 30 shakes/second for 2 min. Homogenates were clarified by centrifugation and virus titer in the supernatant determined by plaque assay. Mosquito saliva was amplified in Vero cells and cytopathic effect assessed 5 days p.i. following fixation and staining with crystal violet.

For detection of DVG or WT RNA from whole mosquitoes or mosquito parts, RNA extracted from homogenates was used for reverse transcription using random primers (Maxima first-strand cDNA synthesis kit, Thermo Fisher). PCRs were then carried out (DreamTaq [Thermo Fisher] or Phusion [NEB]) using the PCR-WT/DVG set of primers (Supplementary Table 3) that detect both WT and DVG RNA, but with different amplicon sizes.

### Human blood and ethics statement

Human blood used to feed mosquitoes was obtained from healthy volunteer donors. Healthy donor recruitment was organized by the local investigator assessment using medical history, laboratory results, and clinical examinations. Biological samples were supplied through participation of healthy volunteers at the ICAReB biobanking platform (BB-0033-00062/ICAReB platform/Institut Pasteur, Paris/BBMRI AO203/[BIORESOURCE]) of the Institut Pasteur to the CoSImmGen and Diagmicoll protocols, which have been approved by the French Ethical Committee (CPP) Ile-de-France I. The Diagmicoll protocol was declared to the French Research Ministry under the reference: DC 2008–68 COL 1.

### Statistical analyses

All data were analyzed using Prism 7 software (GraphPad) and are presented as means ± standard deviation (SD) unless indicated otherwise. For highly skewed data, statistics were carried out using log-transformed data to improve the homoscedasticity of the data. The chosen method of statistical analysis is described in each figure legend. No statistical significance is indicated if *p* is >0.05.

### Reporting summary

Further information on research design is available in the Nature Research Reporting Summary linked to this article.

## Supplementary information

Supplementary Information

Description of Additional Supplementary Files

Supplementary Data 1

Supplementary Data 2

Supplementary Data 3

Supplementary Data 4

Reporting Summary

## Data Availability

The NGS datasets generated and analyzed during the current study have been deposited in the NCBI Sequence Read Archive under accession number PRJNA703982. Source data are provided with this paper.

## Source data

Source Data

## Code availability

The code used for the computational determination of neighborhoods enriched with deletions is available at https://github.com/sharvadim07/DeletionsIslands^61^.
