# Supplementary material for: Defective viral genomes as therapeutic interfering particles against flavivirus infection in mammalian and mosquito hosts
Source: Nat Commun. 2021 Apr 16;12:2290. doi: 10.1038/s41467-021-22341-7 (PMC8052367; doi:10.1038/s41467-021-22341-7)
Supplement: Supplementary file 1 — Supplementary Information [file 41467_2021_22341_MOESM1_ESM.pdf]

High fitness deletion hotspots in defective viral genomes enable the development of therapeutic interfering particles against flavivirus infection in mammalian and mosquito hosts.

Supplementary Information

Supplementary Table 1. Primers used in this study. Underlined nucleotides depict a T7 promoter sequence. Y refers to a C or T.

Primer names	Primer sequence (5’→3’)	Primer use
hGAPDH-F	GTCTCCTCTGACTTCAACAGCG	qPCR of human GAPDH
hGAPDH-R	ACCACCCTGTTGCTGTAGCCAA	
hIFN-α1-F	AGAAGGCTCCAGCCATCTCTGT	qPCR of human IFN-α1
hIFN-α1-R	TGCTGGTAGAGTTCGGTGCAGA	
hIFN-β1-F	GTCAGAGTGGAAATCCTAAG	qPCR of human IFN-β1
hIFN-β1-R	ACAGCATCTGCTGGTTGAAG	
hIFN-γ-F	CTAATTATTCGGTAACATTCGGTAA	qPCR of human IFN-γ
hIFN-γ-R	ACAGTTCAGCCATCACTTGGA	
hIFN-λ1-F	AACTGGGAAGGGCTGCCACATT	qPCR of human IFN-λ1
hIFN-λ1-R	GGAAGACAGGAGAGCTGCAACT	
hRIG-I-F	CACCTCAGTTGCTGATGAAGG	qPCR of human RIG-I
hRIG-I-R	GTCAGAAGGAAGCACTTGCTACC	
T7-Ago2-F	TAATACGACTCACTATAGGGAGAGCCCTCAACAAGAAGCACCCTGATTCATGCAC	Amplification of Ago-2 flanked by A T7 promoter to generate dsRNA
T7-Ago2-R	TAATACGACTCACTATAGGGAGAGCGTTGATCTTCAACCAAATGTTGCTG	
Ago2-qPCR-F	GATTCCGGATCGAGTACCGGAATG	RT-qPCR of *Ae. Albopictus* Ago-2 mRNA
Ago2-qPCR-R	GTCATCTCTGTACCGTTTTCCAG	
Actin-qPCR-F	AAGGCTAACCGYGAGAAGATGAC	RT-qPCR of *Ae. Albopictus* actin mRNA
Actin-qPCR-F	GATTGGGACAGTGTGGGAGAC	
WT-qPCR-F	TCGTTGCCCAACACAAG	RT-qPCR of WT mRNA
WT-qPCR-R	CCACTAATGTTCTTTTGCAGACAT	
WT-qPCR-probe	[6-FAM]-GCCTACCTTGACAAGCAATCAGACACTCA-IABkFQ	
DVG-A-qPCR-F	GGAAGGCCATTTCGTTTGC	RT-qPCR of DVG-A mRNA
DVG-A-qPCR-R	GATTTCAAGCTCTTCACTGTGC	
DVG-A-qPCR probe	[6-FAM]-ACAAGTGCCACGTAAACACCAGAGA-IABkFQ	
mGAPDH-F	AGCAAGGACACTGAGCAAGAG	RT-qPCR of mouse GAPDH
mGAPDH-R	GGGTCTGGGATGGAAATTGTG	
PCR-WTDVG-A -F	CTTGCTGCCATGTTGAG	PCR amplification of WT and DVG-A from cDNA
PCR-WTDVG-A -R	GATCATGACTACCAGCAC	

Supplementary Figures

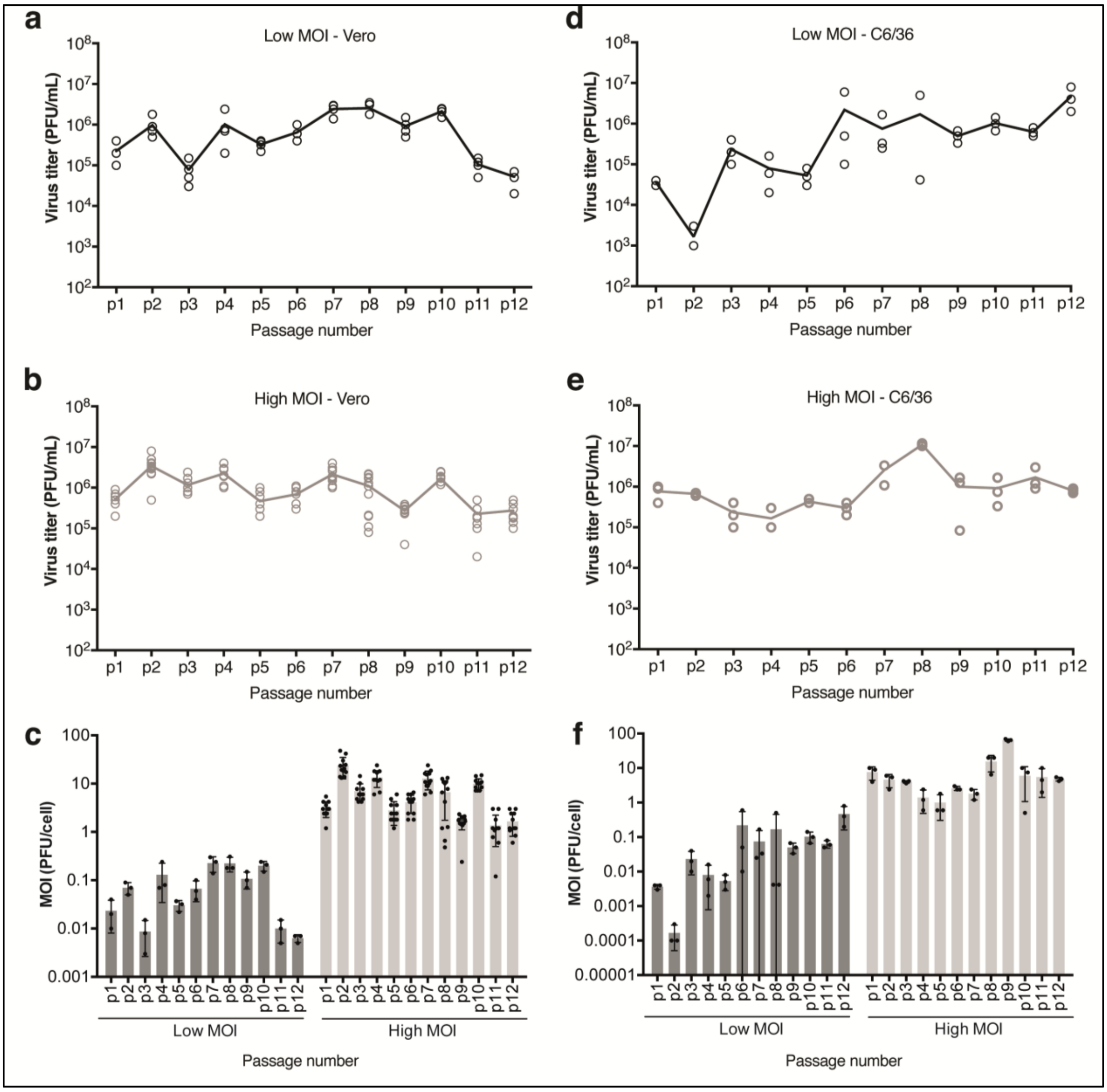

Supplementary Figure 1. Zika virus passages in Vero and C6/36 cell lines. Zika virus was passaged blindly twelve times in Vero (a-c) or C6/36 cells (d-f), at a low MOI (n=3) or high MOI (n=12 and n=3, for Vero and C6/36 cells, respectively). Virus titers in each passage are shown. The MOI used in each passage was confirmed by back-calculation, taking into account the virus titer in the previous passage and number of cells used for Vero (c) or C6/36 (f) cell infections, mean titer and SD are shown. Source data are provided as a Source Data file.

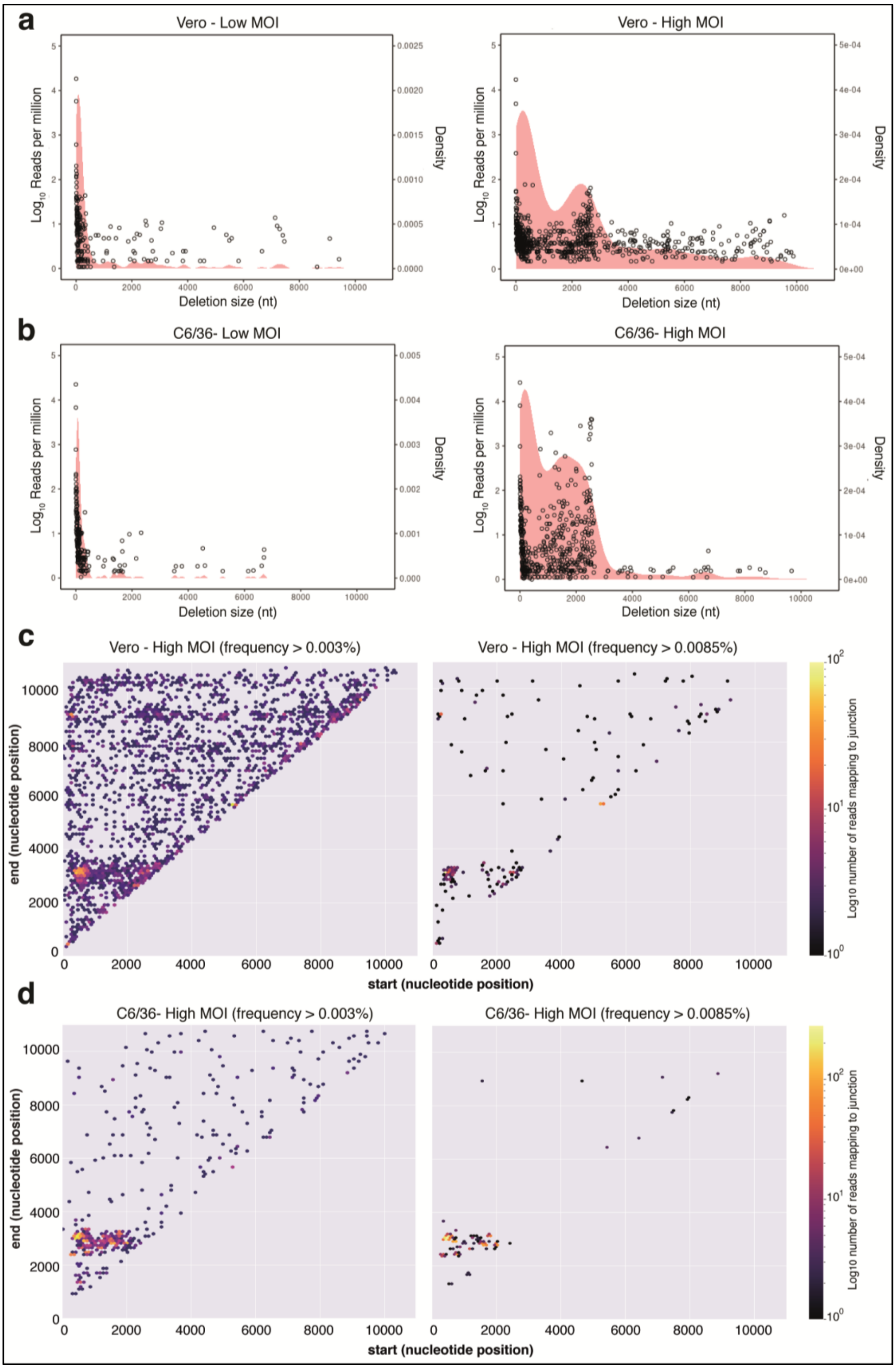

Supplementary Figure 2. Zika virus large deletions are more common under high MOI passaging conditions. (a-b) Mean number of reads supporting a deletion per million reads that align to the virus genome (RPM) across replicates are shown, overlayed with a Kernel density estimation plot and plotted relative to the deletion size, for all deletions identified in Vero (a) or C6/36 (b) cell passaging. **(c-d)** Hexbin plots showing the number of reads mapping deletion of defined start and end nucleotide positions within each hexagon, for high

MOI passages carried out in Vero (c) or C6/36 (d) cells. Deletions with a frequency of > 0.003% are shown on the left panels, and deletions with frequency > 0.0085% are shown on the right panels. Source data are provided in the Supplementary Data Table 1 and 2 for Vero and C6/36 data, respectively.

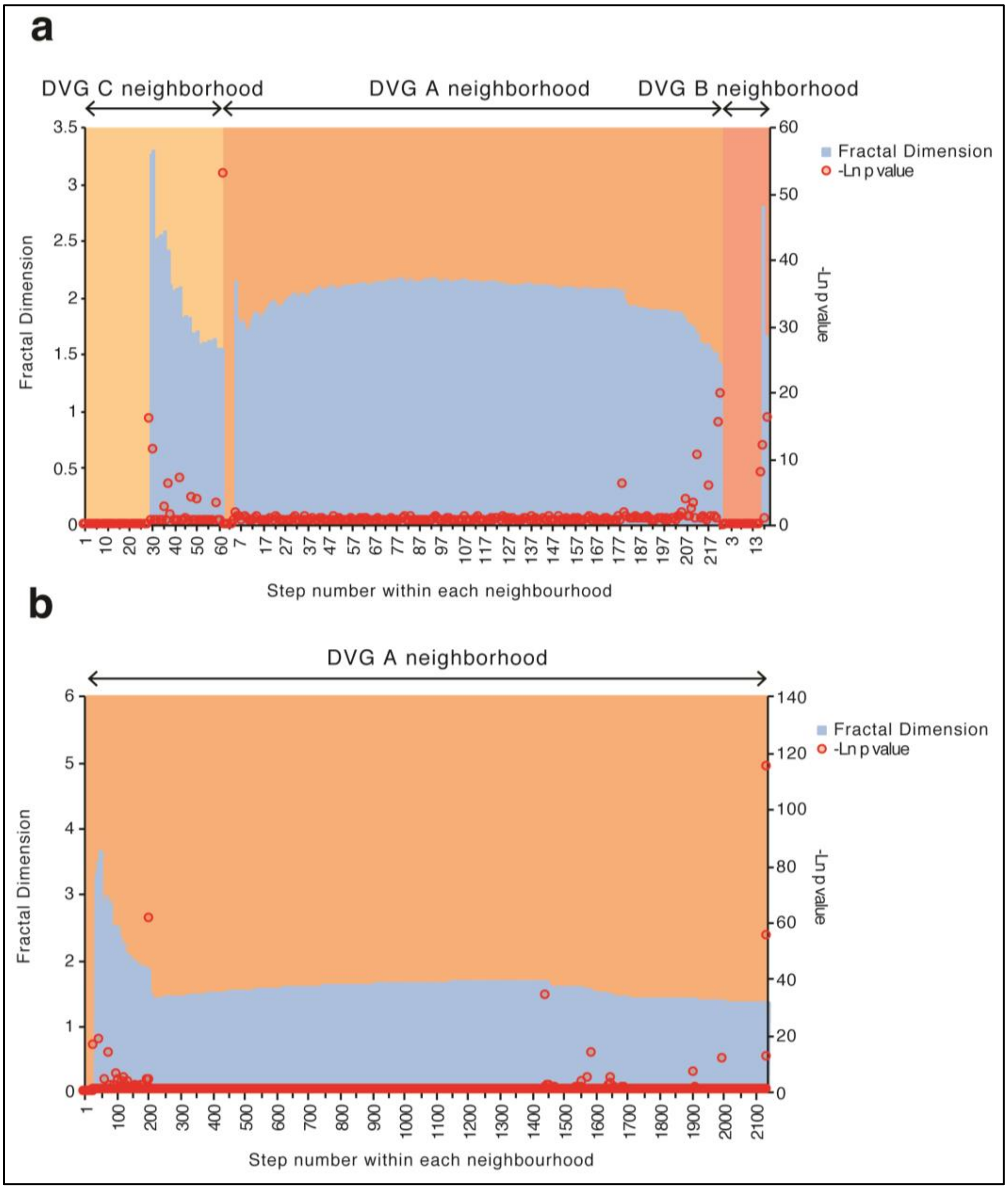

Supplementary Figure 3. Fractal dimensions for neighborhoods identified in Vero and C6/36 cell lines. (a-b) Fractal dimension and *p*-values of enrichment for each step performed in the sequential nested expansions of a neighborhood are shown for Vero (a) and C6/36 (b) cell lines. In (a), the sequential nested fractal dimensions and *p*-values of enrichment are presented for the three neighborhoods identified. In (b) the sequential nested fractal dimensions and *p*-values of enrichment are presented for the sole identified neighborhood. The Poisson p-value shown is the one-side probabilistic measure for deviation from uniformity of the data-point distribution in a volume, and was derived as described in the Methods section. Source data are provided in the Supplementary Data Table 1 and 2 for Vero and C6/36 data, respectively.

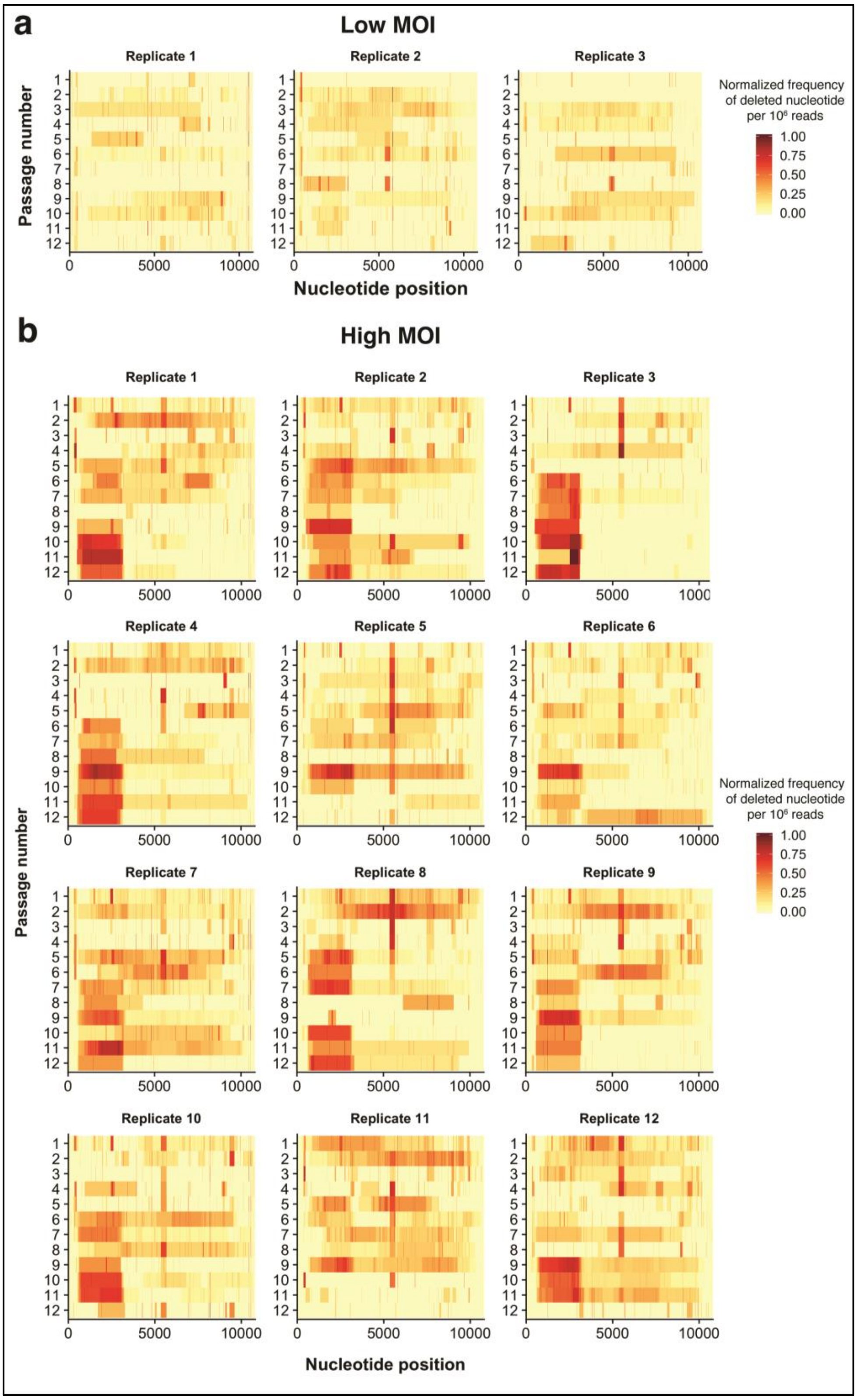

Supplementary Figure 4. Heatmaps of Zika virus deletions identified in all replicates of Vero cell passages. Virus populations in each passage and replicate carried out in Vero cells were sequenced and deletions identified. Heatmaps of deletions identified in Vero cells at low **(a)** or high **(b)** MOI for all replicates are shown. Heatmaps denote the relative number of reads supporting a deletion per million reads that align to the virus genome (reads per million, RPM) at each nucleotide position. Deletion frequencies were normalized relative to the highest and lowest values in each replicate throughout the passages. Source data are provided in the Supplementary Data Table 1 and 2 for Vero and C6/36 data, respectively.

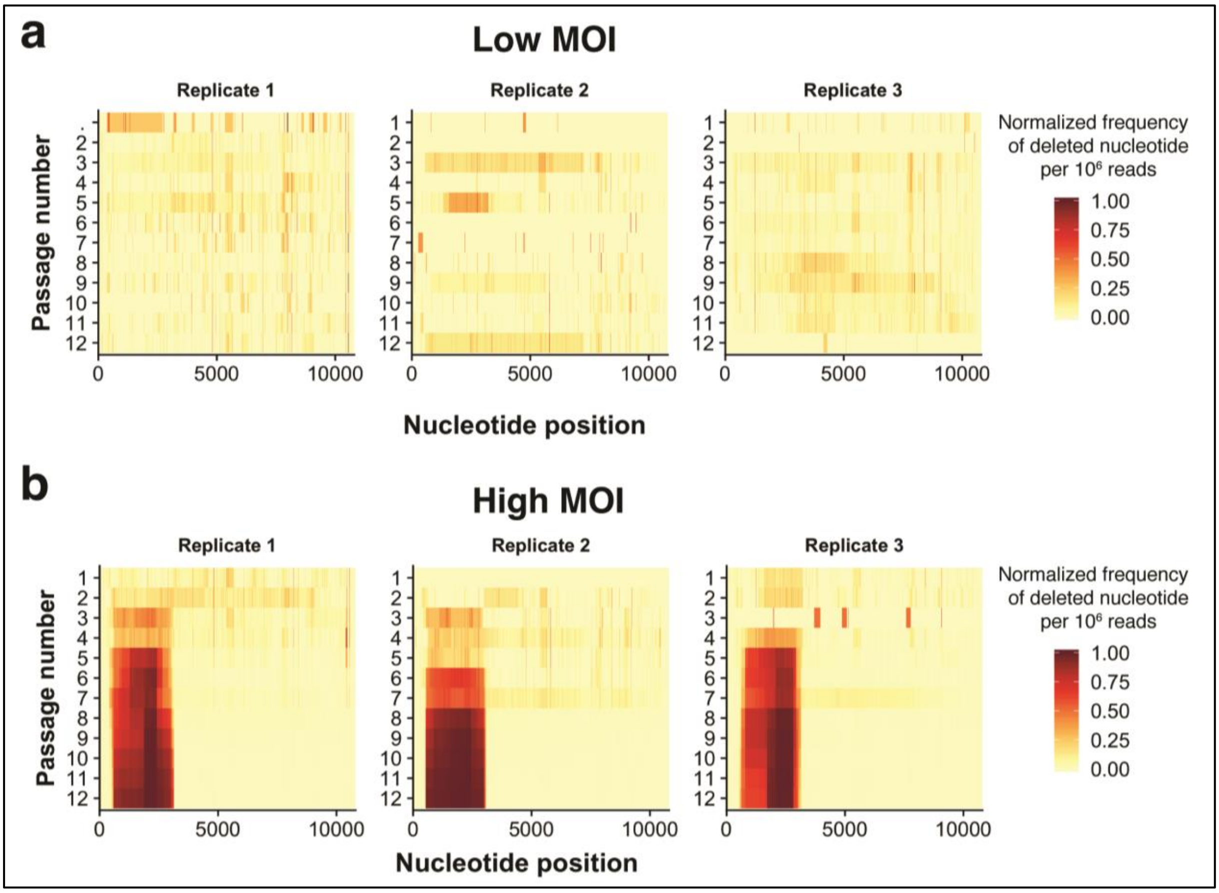

Supplementary Figure 5. Heatmaps of Zika virus deletions identified in all replicates of C6/36 cell passages. Virus populations in each passage and replicate carried out in C6/36 cells were sequenced and deletions identified. Heatmaps of deletions identified in C6/36 cells at low **(a)** or high **(b)** MOI for all replicates are shown. Heatmaps denote the relative number of reads supporting a deletion per million reads that align to the virus genome (RPM) at each nucleotide position. Deletion frequencies were normalized relative to the highest and lowest values in each replicate throughout the passages. Source data are provided in the Supplementary Data Table 1 and 2 for Vero and C6/36 data, respectively.

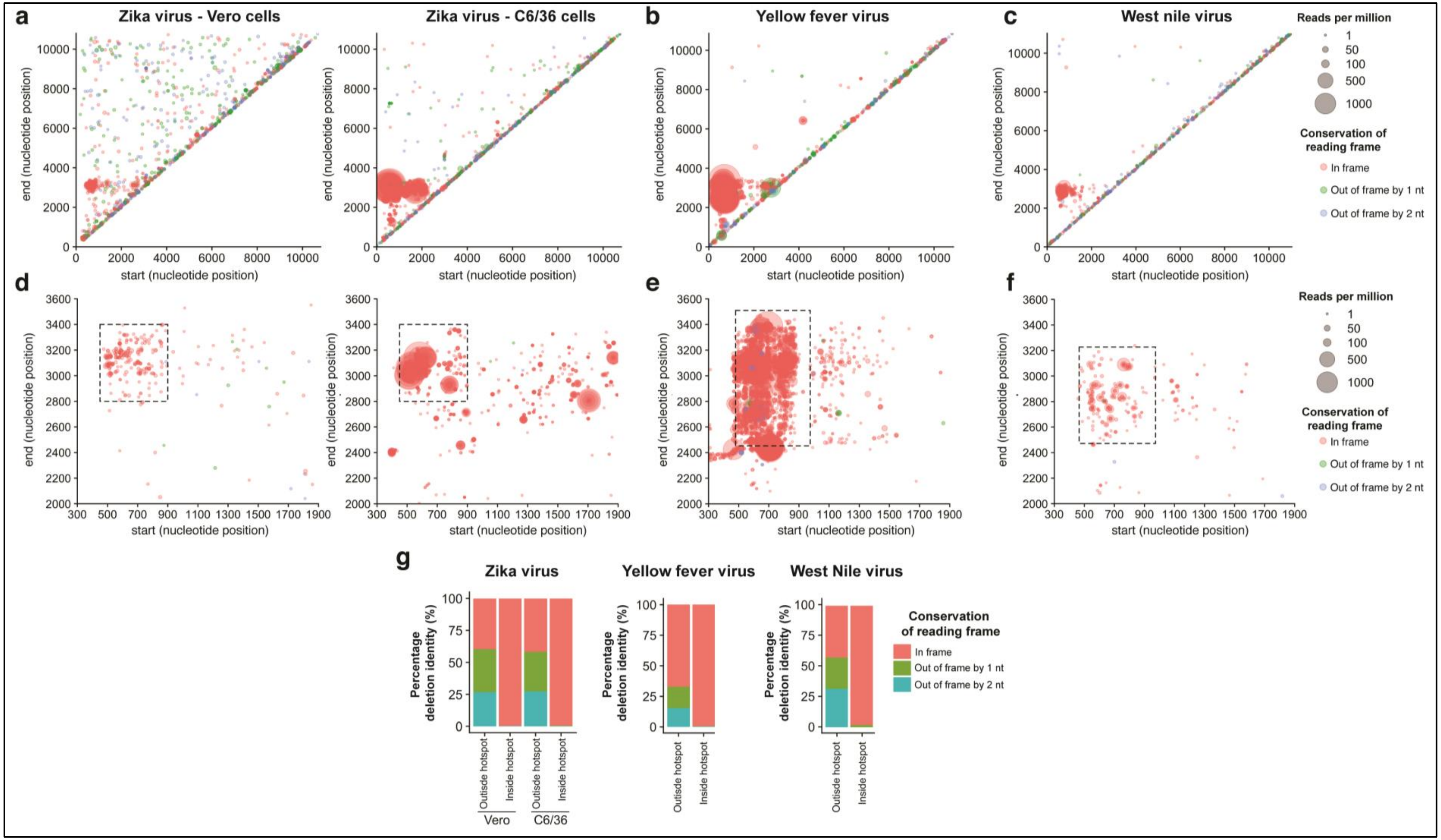

Supplementary Figure 6. Flavivirus deletion hotspots encompass deletions that conserve the open reading frame. (a-c) Start and end positions of each deletion event identified following high MOI passaging of: **(a)** Zika virus in Vero (left panel) or C6/36 (right panel), **(b)** Yellow fever virus in SW-13 cells, or **(c)** West Nile virus in C6/36 cells. Each deletion event iss depicted as a single dot and classified according to the conservation of the open reading frame following the deletion: unchanged ORF (orange), frame-shift by one nucleotide (green), or two nucleotides (blue). The size of each dot is relative to the sum of the reads per million of each deletion in each passage and replicate. Darker areas represent areas in which deletion events overlap. **(d-f)** Close-up analysis of deletions in the region of interest are shown in (d), (e) and (f) or Zika virus, Yellow fever virus or West Nile virus, respectively. The Zika virus DVG-A hotspot and hotspots for Yellow fever virus and West Nile

virus are depicted as the area inside the dotted rectangle. **(g)** Percentage deletion identity according to the conservation of the open reading frame following each deletion event (deletion events of >10nt in length were considered). Percentage deletion identity inside the hotspot and in the rest of the genome is calculated based on deletion diversity: the number of deletions identified which are in frame, out of frame by 1 nt or out of frame by 2 nucleotides, relative to the total number of deletions included in the cluster, or excluded from the cluster. Source data are provided in the Supplementary Data Table 1 and 2 for Vero and C6/36 data, respectively and in Supplementary Data Table 3 and 4 for Yellow fever virus and West Nile virus, respectively.

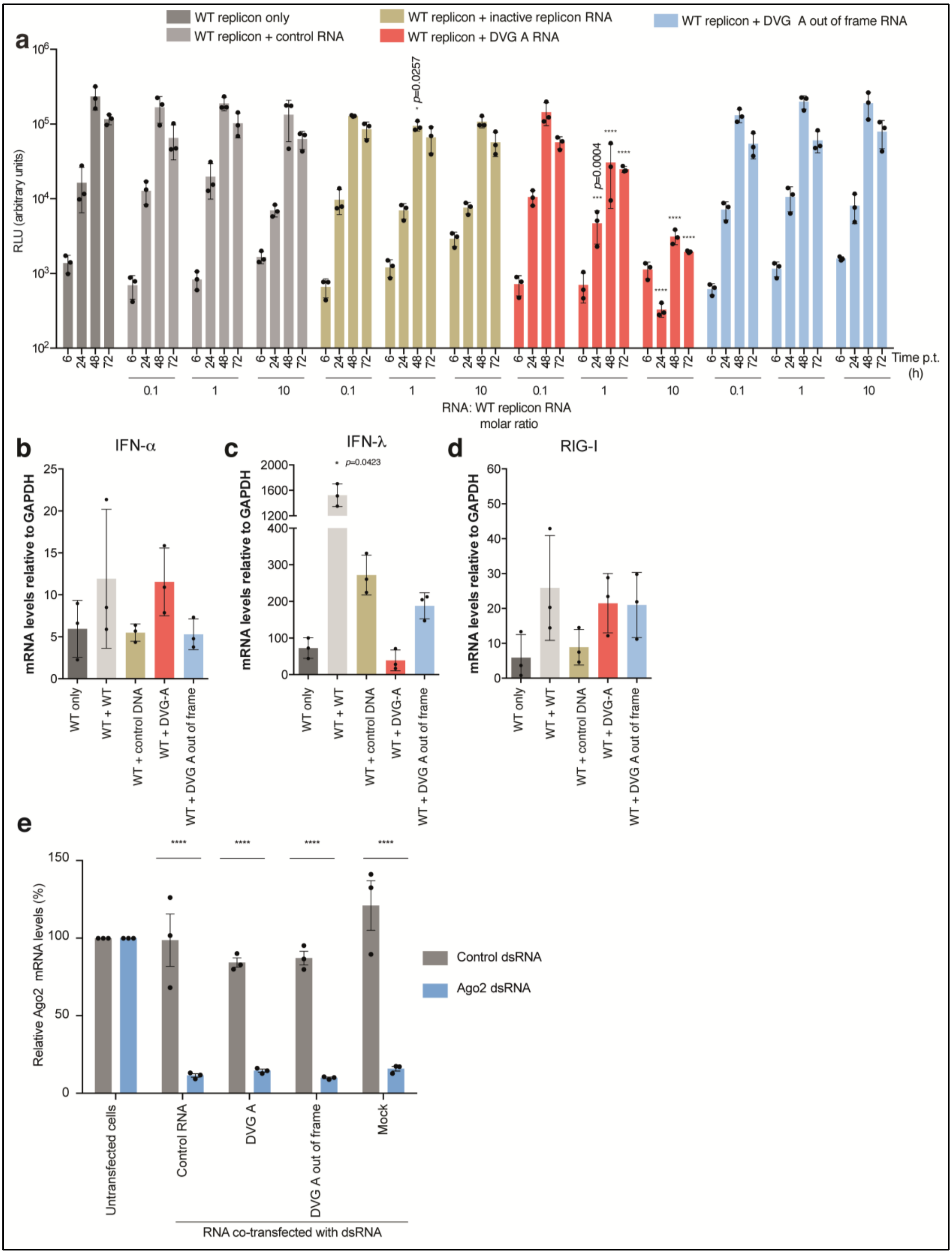

Supplementary Figure 7. DVG-A-induced inhibition in vertebrate and invertebrate environments. (a) WT replicon assay with increasing amounts of DVG RNA. Vero cells were transfected with WT replicon RNA complemented with increasing molar ratios of control control, inactive replicon, DVG-A or DVG-A out-of-frame RNA. Replicon activity was measured at the indicated time points p.t. $p \leq 0.0001$ (by two-way ANOVA with Dunnet's multiple comparison, as compared to cells transfected with WT replicon only). **(b-d)** mRNA levels of IFN-alpha (b), IFN-lambda (c) or RIG-I (d), relative to those of GAPDH in HEK-

293T cells transfected with 10:1 DVG:WT molar ratios, 72h p.t. IFN-alpha and RIG-I levels were compared to WT-only transfected cells by one-way ANOVA with Holm-Sidak post-hoc test. IFN-lambda levels were compared to WT-only transfected cells by Kruskal-Wallis with Dunn post-hoc (*:p=0.0423) because of non-normal distribution. **(e)** Ago-2 knock-down in U4.4 cells. Ago-2 mRNA levels (relative to actin) in transfected and infected cells at the time of virus harvest in Fig 3h are shown. Ago-2 and actin levels were normalized using un-transfected cells as a reference. ****= $p \leq 0.0001$ (two-way ANOVA with Dunnet's multiple comparison). All graphs show the mean and SD; n=3 per group for a representative experiment of three. Control RNA: pTri Xef RNA. Source data are provided as a Source Data file.

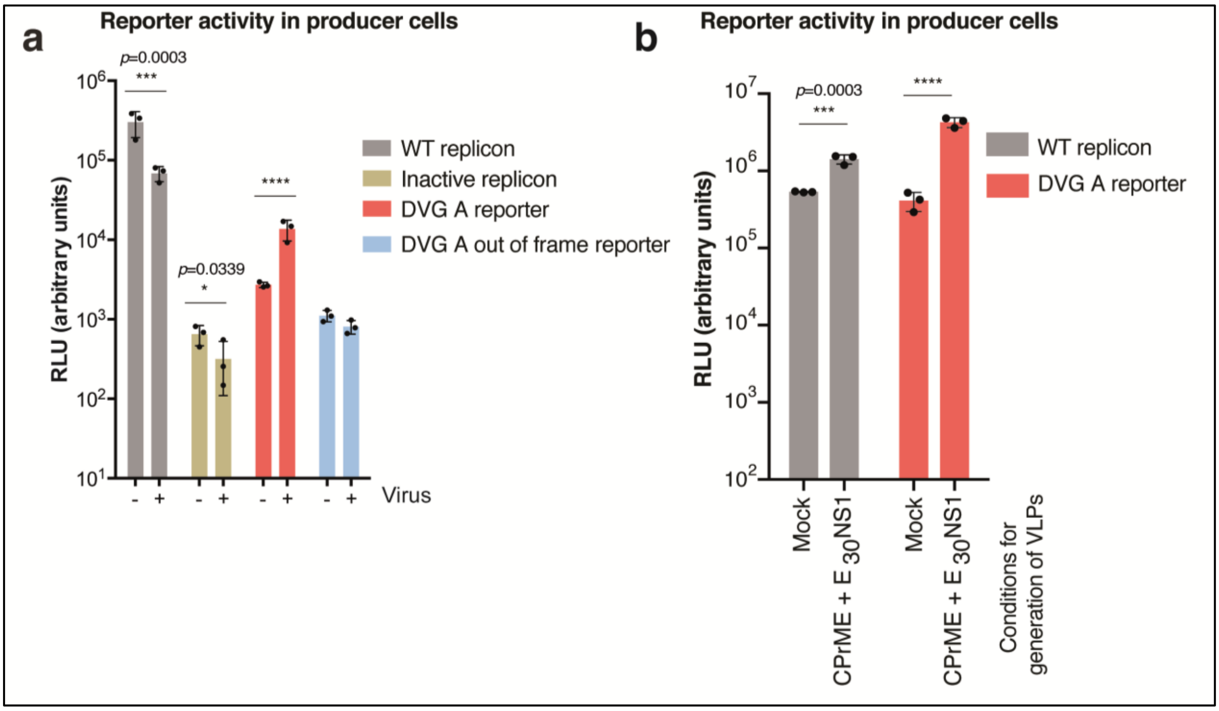

Supplementary Figure 8. Reporter activity in producer cells of packaging assays. (a) Infected or uninfected Vero cells were transfected with WT replicon, inactive replicon, DVG-A reporter or DVG-A out-of-frame reporter RNA. 48h post transfection, the cell supernatant was collected from producer cells, depleted of naked RNA, and used to infect naïve recipient cells (shown in Fig 4a). Reporter activity in producer cells is shown. ****= $p \leq 0.0001$ (by two-way ANOVA with Dunnet's multiple comparison, as compared to uninfected cells). **(b)** VLP assays using WT and DVG-A reporter. Donor HEK293T cells were transfected with WT or DVG-A reporter with or without (mock) CPrME and E_{30}-NS1. The cell culture supernatant was treated with nuclease and used to infect naïve cells. Replicon activity (72h p.t.) in producer cells is shown. ****= $p \leq 0.0001$ (by two-way ANOVA with Dunnet's multiple comparison, as compared to mock conditions). All graphs show the mean and SD; n=3 per group for a representative experiment of three. Source data are provided as a Source Data file.

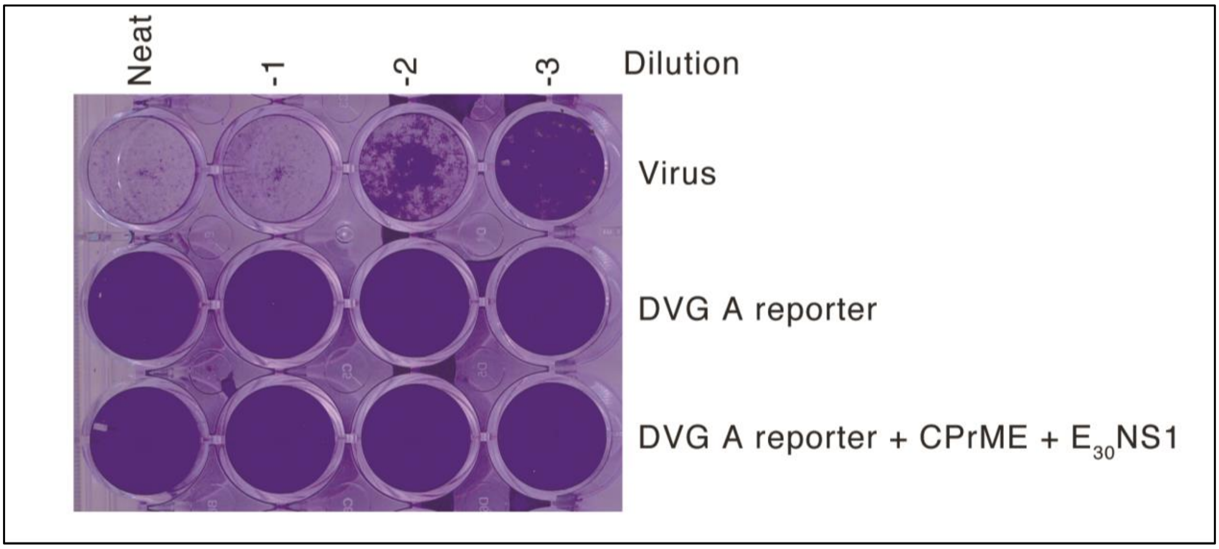

Supplementary Figure 9. Plaque assay to confirm lack of infectious virus in VLP preparations. Plaque assays were carried out with VLP preparations to confirm that no infectious virus was present. The conditions used to generate VLPs (either only with DVG-A reporter, or complemented with structural proteins and NS1) are shown, with wild-type virus serving as a positive control.

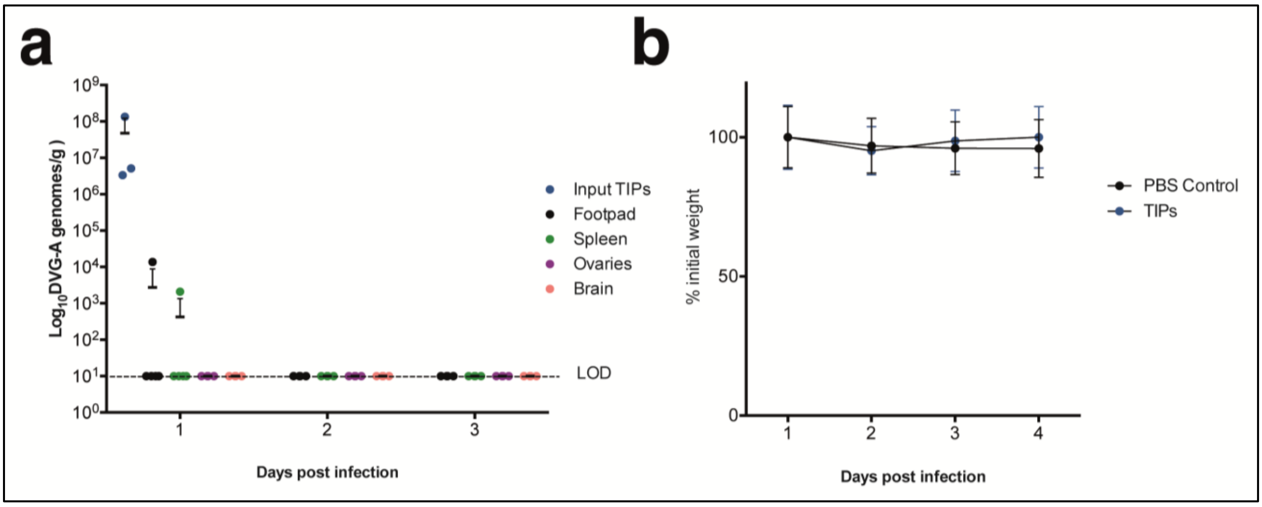

Supplementary Figure 10. Zika virus DVG-A-carrying TIPs degrade in the absence of WT virus and do not pose adverse effects. 4-6 week old AG129 were administered DVG-A-carrying TIPs via a footpad injection. **(a)** DVG-A amounts in organs collected at 1, 2 or 3 days post administration are shown, as measured by RT-qPCR (n=5). DVG-A RNA levels in TIPs used as an input for administration were measured as a control (n=3). **(b)** Weight change in mice administered DVG-A carrying TIPs (n=5). In all graphs, mean and SD are shown. Source data are provided as a Source Data file.

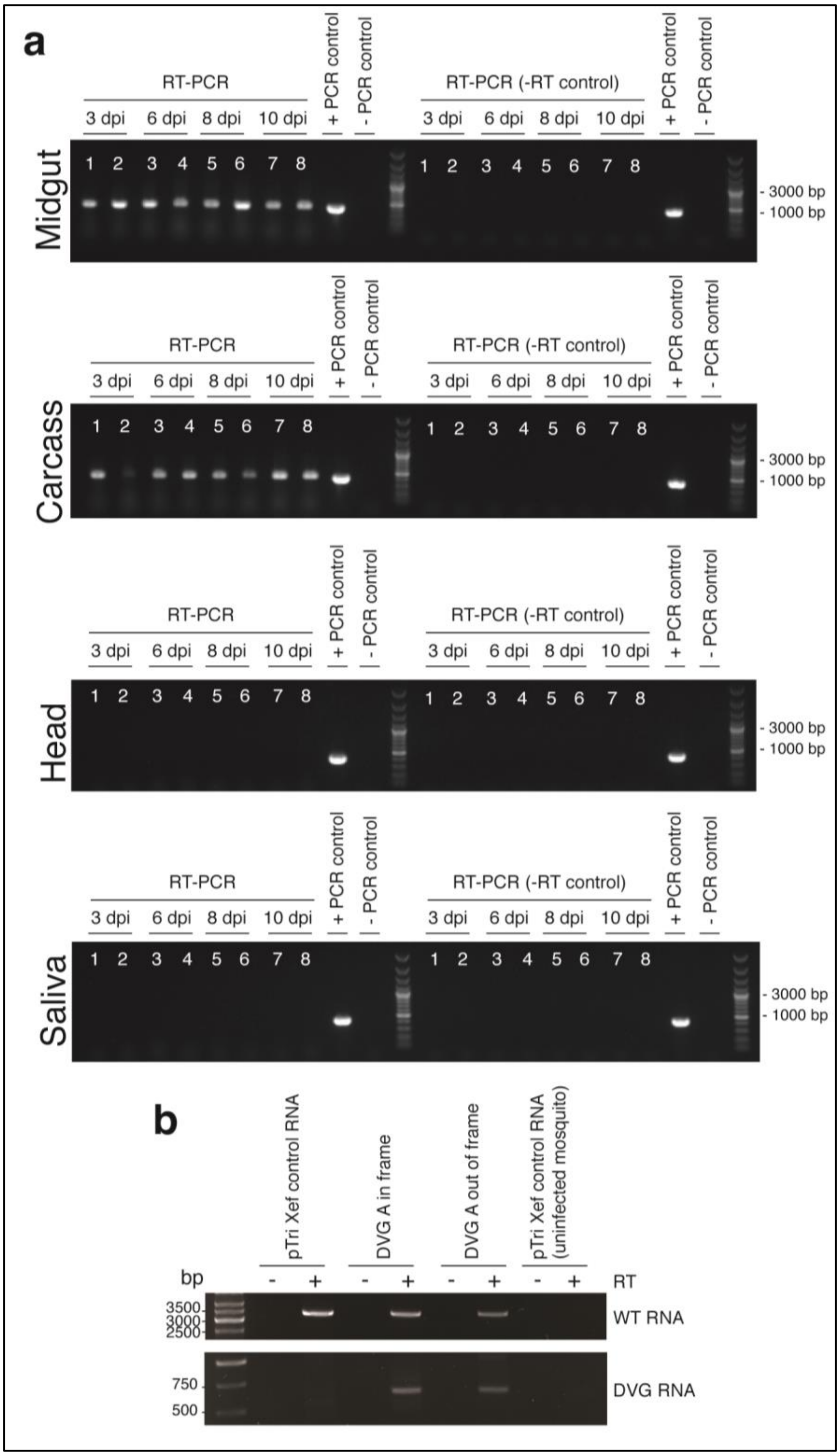

Supplementary Figure 11. Longevity of DVG-A RNA in mosquitoes. (a) *Ae. aegypti* mosquitoes were *in vivo* transfected with a transfection mix containing equimolar solutions of DVG-A or DVG-A out-of-frame RNA. At the indicated times post-transfection, 5 mosquitoes were salivated and midgut, carcass, and head dissected. RNA was extracted from homogenates of a pool of the 5 mosquitoes and RT-PCR specific for DVG-A was performed. Lanes 1, 3, 5 and 7 = DVG-A out-of-frame, Lanes 2, 4, 6 and 8 = DVG-A. **(b)** RT-PCR for whole mosquitoes transfected with control, DVG-A or DVG-A out-of-frame RNA and infected following a Zika virus bloodmeal. A single whole mosquito from the experiment was homogenized 13 days p.i. and RNA from the homogenate used for RT-PCR for the detection of WT RNA (higher molecular weight band) and DVG-A RNA (lower molecular weight band). bp = basepairs. Results from a representative experiment out of two are shown in both panels.

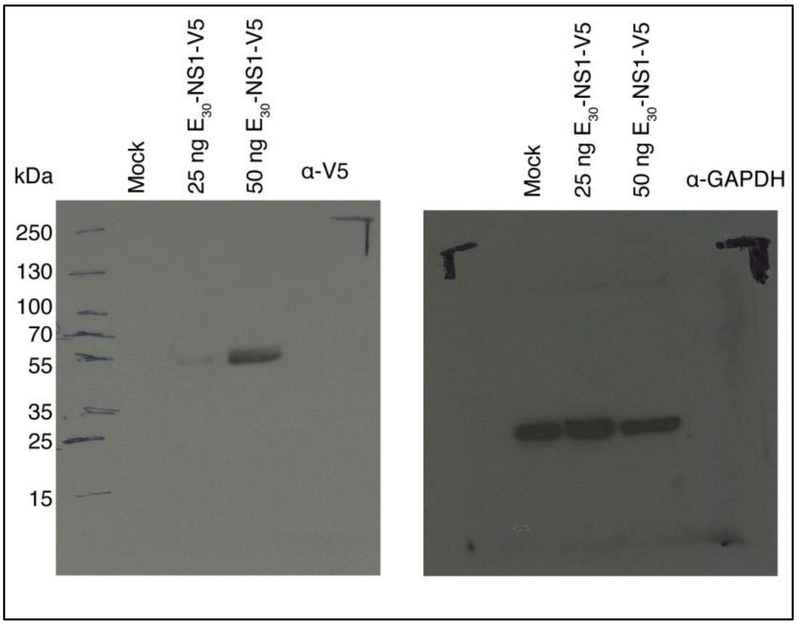

Supplementary Figure 12. Western blot of cells transfected with V5-tagged E_{30}-NS1. Uncropped image of western blot corresponding to Fig 2e.
