# Supplementary material for: Defective viral genomes as therapeutic interfering particles against flavivirus infection in mammalian and mosquito hosts
Source: Nat Commun. 2021 Apr 16;12:2290. doi: 10.1038/s41467-021-22341-7 (PMC8052367; doi:10.1038/s41467-021-22341-7)
Supplement: Supplementary file 2 — Description of Additional Supplementary Files [file 41467_2021_22341_MOESM2_ESM.pdf]

Description of Additional Supplementary Files

File Name: Supplementary Data 1
Description: **Zika virus deletions identified in Vero cell passages.** All start and stop positions for all deletions are listed 'Start' refers to the first deleted nucleotide, 'Stop' refers to the first nucleotide following the deletion. 'Del.size' refers to the deletion size in nucleotides. 'OF.by' refers to the conservation of the open reading frame following the deletion (0 for no change, 1 for onenucleotide frame-shift, 2 for two-nucleotide frame-shift). 'DVG.size' refers to the resulting deletion-bearing genome size . 'Passage', 'Replicate' and 'MOI' refer to passaging conditions of the sample where the deletion was found (passage number, replicate and multiplicity of infection, respectively). 'RPM' refers to deletion reads per million

File Name: Supplementary Data 2
Description: **Zika virus deletions identified in C6/36 cell passages.** All start and stop positions for all deletions are listed 'Start' refers to the first deleted nucleotide, 'Stop' refers to the first nucleotide following the deletion. 'Del.size' refers to the deletion size in nucleotides. 'OF.by' refers to the conservation of the open reading frame following the deletion (0 for no change, 1 for onenucleotide frame-shift, 2 for two-nucleotide frame-shift). 'DVG.size' refers to the resulting deletion-bearing genome size . 'Passage', 'Replicate' and 'MOI' refer to passaging conditions of the sample where the deletion was found (passage number, replicate and multiplicity of infection, respectively). 'RPM' refers to deletion reads per million.

File Name: Supplementary Data 3
Description: **Yellow fever virus deletions identified in SW-13 cell passages.** All start and stop positions for all deletions are listed 'Start' refers to the first deleted nucleotide, 'Stop' refers to the first nucleotide following the deletion. 'Del.size' refers to the deletion size in nucleotides. 'OF.by' refers to the conservation of the open reading frame following the deletion (0 for no change, 1 for one-nucleotide frame-shift, 2 for twonucleotide frame-shift). 'DVG.size' refers to the resulting deletion- bearing genome size . 'Passage', 'Replicate' and 'MOI' refer to passaging conditions of the sample where the deletion was found (passage number, replicate and multiplicity of infection, respectively). 'RPM' refers to deletion reads per million.

File Name: Supplementary Data 4
Description: **West Nile virus deletions identified in C6/36 cell passages.** All start and stop positions for all deletions are listed 'Start' refers to the first deleted nucleotide, 'Stop' refers to the first nucleotide following the deletion. 'Del.size' refers to the deletion size in nucleotides. 'OF.by' refers to the conservation of the open reading frame following the deletion (0 for no change, 1 for one-nucleotide frame-shift, 2 for twonucleotide frame-shift). 'DVG.size' refers to the resulting deletionbearing genome size . 'Passage', 'Replicate' and 'MOI' refer to passaging conditions of the sample where the deletion was found (passage number, replicate and multiplicity of infection, respectively). 'RPM' refers to deletion reads per million.
