# Supplementary material for: Defective viral genomes as therapeutic interfering particles against flavivirus infection in mammalian and mosquito hosts
Source: Nat Commun. 2021 Apr 16;12:2290. doi: 10.1038/s41467-021-22341-7 (PMC8052367; doi:10.1038/s41467-021-22341-7)
Supplement: Supplementary file 7 — Reporting Summary [file 41467_2021_22341_MOESM7_ESM.pdf]

nature research

Corresponding author(s): Marco Vignuzzi

Last updated by author(s): Mar 9, 2021

Reporting Summary

Nature Research wishes to improve the reproducibility of the work that we publish. This form provides structure for consistency and transparency in reporting. For further information on Nature Research policies, see our Editorial Policies and the Editorial Policy Checklist.

Statistics

For all statistical analyses, confirm that the following items are present in the figure legend, table legend, main text, or Methods section.

n/a	Confirmed	
☐	☒	The exact sample size (*n*) for each experimental group/condition, given as a discrete number and unit of measurement
☐	☒	A statement on whether measurements were taken from distinct samples or whether the same sample was measured repeatedly
☐	☒	The statistical test(s) used AND whether they are one- or two-sided<br>*Only common tests should be described solely by name; describe more complex techniques in the Methods section.*
☐	☒	A description of all covariates tested
☐	☒	A description of any assumptions or corrections, such as tests of normality and adjustment for multiple comparisons
☐	☒	A full description of the statistical parameters including central tendency (e.g. means) or other basic estimates (e.g. regression coefficient) AND variation (e.g. standard deviation) or associated estimates of uncertainty (e.g. confidence intervals)
☐	☒	For null hypothesis testing, the test statistic (e.g. *F*, *t*, *r*) with confidence intervals, effect sizes, degrees of freedom and *P* value noted<br>*Give P values as exact values whenever suitable.*
☒	☐	For Bayesian analysis, information on the choice of priors and Markov chain Monte Carlo settings
☒	☐	For hierarchical and complex designs, identification of the appropriate level for tests and full reporting of outcomes
☒	☐	Estimates of effect sizes (e.g. Cohen's *d*, Pearson's *r*), indicating how they were calculated

Our web collection on statistics for biologists contains articles on many of the points above.

Software and code

Policy information about availability of computer code

Data collection	n/a
Data analysis	BBMap suite version 38.06 (https://sourceforge.net/projects/bbmap/) was used for NGS analysis and is described in the methods section. Codes for algorithms used in this study were deposited in GitHub and are referenced in the manuscript.

For manuscripts utilizing custom algorithms or software that are central to the research but not yet described in published literature, software must be made available to editors and reviewers. We strongly encourage code deposition in a community repository (e.g. GitHub). See the Nature Research guidelines for submitting code & software for further information.

Data

Policy information about availability of data

All manuscripts must include a data availability statement. This statement should provide the following information, where applicable:

- Accession codes, unique identifiers, or web links for publicly available datasets
- A list of figures that have associated raw data
- A description of any restrictions on data availability

The NGS datasets generated and analyzed during the current study have been deposited in the NCBI Sequence Read Archive under accession number PRJNA703982. Source data for all figures are provided with this paper.

Field-specific reporting

Please select the one below that is the best fit for your research. If you are not sure, read the appropriate sections before making your selection.

☒ Life sciences ☐ Behavioural & social sciences ☐ Ecological, evolutionary & environmental sciences

For a reference copy of the document with all sections, see nature.com/documents/nr-reporting-summary-flat.pdf

Life sciences study design

All studies must disclose on these points even when the disclosure is negative.

Sample size	For mouse experiments, advice was sought from statisticians at the Institut Pasteur Informatics Hub to perform power tests based on effects expected to result in a 10-fold or greater decrease in viral titers for a DVG-treated group compared to a control group, based on titers and variance from previous studies involving Zika virus in this mouse model.
Data exclusions	No data were excluded.
Replication	Experiments were successfully replicated three times.
Randomization	Mice were randomly assigned to groups, and once treated, were randomly chosen for virus titer at different time points.
Blinding	Investigators were blinded at data collection (titering of virus in mouse samples) and analysis until all results were completed.

Reporting for specific materials, systems and methods

We require information from authors about some types of materials, experimental systems and methods used in many studies. Here, indicate whether each material, system or method listed is relevant to your study. If you are not sure if a list item applies to your research, read the appropriate section before selecting a response.

Materials & experimental systems

n/a	Involved in the study	
☐	☒	Antibodies
☐	☒	Eukaryotic cell lines
☒	☐	Palaeontology and archaeology
☐	☒	Animals and other organisms
☒	☐	Human research participants
☒	☐	Clinical data
☒	☐	Dual use research of concern

Methods

n/a	Involved in the study	
☒	☐	ChIP-seq
☒	☐	Flow cytometry
☒	☐	MRI-based neuroimaging

Antibodies

Antibodies used	Anti-V5 tag antibody [SV5-Pk1] , mouse, catalogue number, ab27671, lot number not available at the time of completing this summary<br>Mouse IgG HRP Linked Whole Ab, Cytiva (previously GE life sciences), NA931-1ML
Validation	The primary antibody has been referenced in 79 publications, as detailed in the manufacturer's website (https://www.abcam.com/v5-tag-antibody-sv5-pk1-ab27671.html)

Eukaryotic cell lines

Policy information about cell lines

Cell line source(s)	Vero, Vero-E6, HEK-293T, SW-13, C6/36 and U4.4 were all originally derived from ATCC
Authentication	None of the cell lines were authenticated
Mycoplasma contamination	All cell lines used in this study tested negative for mycoplasma contamination
Commonly misidentified lines (See ICLAC register)	*Name any commonly misidentified cell lines used in the study and provide a rationale for their use.*

Animals and other organisms

Policy information about studies involving animals; ARRIVE guidelines recommended for reporting animal research

Laboratory animals	IFNα/β/γR-/- AG129 mice (background strain 129S7/SvEvBrd), males or females, aged between 4-6 weeks were used in this study C57BL/6 mice, strain code 027 (Charles River), females, aged between 4-6 weeks were used in this study. 6-8 days old female Ae. aegypti mosquitoes (1 colony, F7 generation, collected originally in Kamphaeng Phet Province, Thailand) were used in this study.
Wild animals	*Provide details on animals observed in or captured in the field; report species, sex and age where possible. Describe how animals were caught and transported and what happened to captive animals after the study (if killed, explain why and describe method; if released, say where and when) OR state that the study did not involve wild animals.*
Field-collected samples	*For laboratory work with field-collected samples, describe all relevant parameters such as housing, maintenance, temperature, photoperiod and end-of-experiment protocol OR state that the study did not involve samples collected from the field.*
Ethics oversight	All work was performed in compliance with the Animal Committee regulations and guidelines of Institut Pasteur Paris France, under the 2010/63 European Union Council directive. Animal protocols were approved by the Ethics Committee on Animal Experimentation (CETEA) under dossier number dap160116/CHCST 18.176 and the USAMRMC Animal Care and Use Review Office (ACURO), under the protocol number DARPA-5417.04.

Note that full information on the approval of the study protocol must also be provided in the manuscript.
